# TAZ is involved in breast cancer cell migration via regulating actin dynamics

**DOI:** 10.3389/fonc.2024.1376831

**Published:** 2024-05-07

**Authors:** Hong Seok Choi, Hyo-Ju Jang, Mathilde K. Kristensen, Tae-Hwan Kwon

**Affiliations:** ^1^ Department of Biochemistry and Cell Biology, School of Medicine, Kyungpook National University, Taegu, Republic of Korea; ^2^ BK21 FOUR KNU Convergence Educational Program, Department of Biomedical Science, School of Medicine, Kyungpook National University, Taegu, Republic of Korea; ^3^ Faculty of Health, Medicine, Aarhus University, Aarhus, Denmark

**Keywords:** actin, breast cancer, cell migration, small GTPase, TAZ

## Abstract

**Background:**

Cancer metastasis is dependent on cell migration. Several mechanisms, including epithelial-to-mesenchymal transition (EMT) and actin fiber formation, could be involved in cancer cell migration. As a downstream effector of the Hippo signaling pathway, transcriptional coactivator with PDZ-binding motif (TAZ) is recognized as a key mediator of the metastatic ability of breast cancer cells. We aimed to examine whether TAZ affects the migration of breast cancer cells through the regulation of EMT or actin cytoskeleton.

**Methods:**

MCF-7 and MDA-MB-231 cells were treated with siRNA to attenuate TAZ abundance. Transwell migration assay and scratch wound healing assay were performed to study the effects of TAZ knockdown on cancer cell migration. Fluorescence microscopy was conducted to examine the vinculin and phalloidin. Semiquantitative immunoblotting and quantitative real-time PCR were performed to study the expression of small GTPases and kinases. Changes in the expression of genes associated with cell migration were examined through next-generation sequencing.

**Results:**

TAZ-siRNA treatment reduced TAZ abundance in MCF-7 and MDA-MB-231 breast cancer cells, which was associated with a significant decrease in cell migration. TAZ knockdown increased the expression of fibronectin, but it did not exhibit the typical pattern of EMT progression. TGF-β treatment in MDA-MB-231 cells resulted in a reduction in TAZ and an increase in fibronectin levels. However, it paradoxically promoted cell migration, suggesting that EMT is unlikely to be involved in the decreased migration of breast cancer cells in response to TAZ suppression. RhoA, a small Rho GTPase protein, was significantly reduced in response to TAZ knockdown. This caused a decrease in the expression of the Rho-dependent downstream pathway, i.e., LIM kinase 1 (LIMK1), phosphorylated LIMK1/2, and phosphorylated cofilin, leading to actin depolymerization. Furthermore, myosin light chain kinase (MLCK) and phosphorylated MLC2 were significantly decreased in MDA-MB-231 cells with TAZ knockdown, inhibiting the assembly of stress fibers and focal adhesions.

**Conclusion:**

TAZ knockdown inhibits the migration of breast cancer cells by regulating the intracellular actin cytoskeletal organization. This is achieved, in part, by reducing the abundance of RhoA and Rho-dependent downstream kinase proteins, which results in actin depolymerization and the disassembly of stress fibers and focal adhesions.

## Introduction

Breast cancer is one of the most common types of cancer and the second leading cause of death among women (1). Despite advancements in the diagnosis and treatment of early-stage disease, approximately 6-10% of breast cancer patients are diagnosed with metastasis. Furthermore, it has been estimated that around 30% of patients who are initially diagnosed with early-stage breast cancer will ultimately experience recurrent advanced or metastatic disease (2). As opposed to the primary tumor, the cause of mortality in breast cancer patients is predominantly the metastatic dissemination to multiple organs, including bone, lung, and liver (3, 4). Thus, the heterogeneous nature of breast cancer metastasis makes it challenging to identify risk factors and underlying mechanisms for disease progression and define appropriate treatments for each breast cancer patient (5).

Tumor metastasis involves a complex set of sequential events. For instance, local invasion into the surrounding tissues, intravasation by infiltrating the blood or lymphatic vessels, and survival as circulating tumor cells (CTCs) in the circulation are all required. In addition, the release of CTCs from the circulatory system, extravasation, adaptation to the microenvironments as disseminated tumor cells, and metamorphosis into metastasis-initiating cells are necessary for the formation of a metastatic lesion (6–9). In particular, cell migration, a fundamental process that is implicated in numerous biological phenomena, is critical for the invasion and metastasis of cancer (10–12).

Several mechanisms could be implicated in cancer cell migration, including epithelial-to-mesenchymal transition (EMT) and actin fiber formation (13). In addition, we previously demonstrated that aquaporin-5 (AQP5) expression is associated with breast cancer cell migration, activated Rac1, and cell detachment and dissemination from migrating cell sheets (14–16). The EMT is known to be engaged in the initiation of cancer cell migration, as it involves the loss of epithelial markers, trans-differentiation into mesenchymal-like cells, and the acquisition of motile and invasive capacities (17, 18). For instance, Snail1, a crucial transcription factor of EMT, is associated with the tumor grade, a high rate of recurrence, and distant or lymph node metastasis of invasive ductal carcinoma (19, 20), suggesting a plausible target for therapeutic intervention. Furthermore, cancer-associated fibroblasts (CAF) assemble a fibronectin-rich extracellular matrix that facilitates CAF-cancer cell interaction and directs cell migration (21).

Importantly, the Hippo signaling pathway, which regulates cell proliferation, differentiation, and apoptosis (22, 23), has emerged as a cancer signaling network in a range of malignancies, including breast cancer (24). Dysregulation of the Hippo pathway is associated with breast cancer metastasis (25–27). Particularly, Bartucci et al. (28) demonstrated that the loss of TAZ (transcriptional co-activator with PDZ-binding motif, also known as WWTR1) in breast cancer stem cells significantly hindered metastasis colonization formation and reduced chemoresistance. Based on these studies, we hypothesized that Hippo signaling might change the ability of cancer cells to metastasize (i.e., cell migration) by controlling EMT or actin fiber formation, in addition to regulating the expression of genes linked to tumor metastasis (29).

This study aimed to investigate the role of TAZ in breast cancer cell migration and its underlying mechanisms. We have focused on studying the alterations in EMT and actin fiber formation in response to TAZ knockdown in breast cancer cells, as these changes may be linked to cancer cell migration. Furthermore, we used next-generation sequencing (NGS) to study alterations in genes associated with TAZ knockdown in breast cancer cells.

## Materials and methods

### Human breast cancer cells

Human breast cancer cell lines [MCF-7 (HTB-22) and MDA-MB-231 (HTB-26)] were purchased from the American Type Culture Collection (ATCC, Manassas, VA). MCF-7 cells were maintained in high glucose Dulbecco’s Modified Eagle’s Medium (DMEM, 25 mM glucose), while MDA-MB-231 cells were cultured in low glucose DMEM (5.5 mM glucose) at 37°C. Both media contained 0.1% penicillin-streptomycin and 10% heat-inactivated fetal bovine serum (FBS). Cells were incubated in a humidified incubator at 37°C and 5% CO_2_. MCF-7 cells and MDA-MB-231 cells were seeded on 6- or 12-well plates and transfected with small interfering RNA (siRNA) specific for TAZ, using siGENOME human WWTR1 siRNA (M-016083-00-0010, Dharmacon; Horizon Discovery, Cambridge, UK), siGENOME human RHOA siRNA (M-003860-03-0010, Dharmacon; Horizon Discovery, Cambridge, UK), and DharmaFECT4 (T-2004-03, Dharmacon; Horizon Discovery, Cambridge, UK). To isolate proteins from the cells for performing immunoblotting, cells were lysed with Laemmli buffer (10 mM Tris-HCl, 1.5% SDS, pH 6.8), containing protease and phosphatase inhibitors (0.4 μg/mL leupeptin, 0.1 mg/mL pefabloc, 1 mM Na_3_VO_4_, 24 mM NaF, and 0.1 μM okadaic acid). Total RNA was extracted using Direct-zol RNA MiniPrep (R2050, Zymo Research, Irvine, CA) following the manufacturer’s instructions for quantitative real-time PCR (qRT-PCR).

### Semiquantitative immunoblotting analysis

Cells were lysed in Laemmli buffer (10 mM Tris-HCl, 1.5% SDS, pH 6.8) The lysates prepared using Laemmli buffer were loaded onto a QIAshredder column (QIAGEN, Hilden, Germany) and centrifuged at 10,000 g for 2 min at room temperature. Total protein concentration was measured using a BCA protein assay kit (Pierce BCA protein assay reagent kit; Pierce, Rockford, IL). Semiquantitative immunoblot analyses were performed, as previously described (16, 30). Primary antibodies used were anti-TAZ (1:1,000, 560235, BD bioscience, Franklin Lakes, NJ), YAP (1:1,000, #14074, Cell signaling Technology, Danvers, MA), RhoA (1:1,000, ab187027, Abcam, Cambridge, UK), RhoC (1:1,000, #3430, Cell signaling Technology, Danvers, MA), ROCK1/2 (1:1,000, ab45171, Abcam, Cambridge, UK), Cdc42 (1:1,000, ab187643, Abcam, Cambridge, UK), Rac1 (1:1,000, 610651, BD bioscience, Franklin Lakes, NJ), vimentin (1:1,000, #5741, Cell signaling Technology, Danvers, MA), Occludin (1:1,000, 71-1500, Invitrogen, Waltham, MA), E-cadherin (1:1,000, 610181, BD bioscience, Franklin Lakes, NJ), N-cadherin (1:1,000, ab18203, Abcam, Cambridge, UK), fibronectin (1:1,000, ab2413, Abcam, Cambridge, UK), Myosin Light Chain Kinase (1:1,000, M7905, Sigma), Myosin Light Chain 2 (1:1,000, #3672, Cell signaling Technology), Phospho-Myosin Light Chain 2 (1:1,000, #3675, Cell signaling Technology), LIMK1 (1:1,000, #3842, Cell signaling Technology), LIMK2 (1:1,000, #3845, Cell signaling Technology), phospho-LIMK (1:1,000, #3841, Cell signaling Technology), cofilin (1:1,000, #5175, Cell signaling Technology), and phospho-cofilin (1:1,000, #3313, Cell signaling Technology). Immunoblots were visualized by horseradish peroxidase-conjugated secondary antibodies (P447 or P448, DAKO, Glostrup, Denmark). The band density was quantitated by Image J (NIH, Bethesda, MD), and the value of densitometry was corrected by the densitometry value of β-actin (1:200,000, A1978, Sigma).

### Quantitative real-time PCR

MDA-MB-231 cells were seeded in 6-well plates and transfected with 25 nM of TAZ-siRNA for 24 h. Total RNA purification was performed by Direct-zol RNA MiniPrep, and total RNA (1 μg) was used to synthesize cDNA with the Takara cDNA synthesis kit (6110A, Takara, Otsu, Shiga, Japan). The relative mRNA expression of TAZ, RhoA, RhoC, ROCK1, ROCK2, Cdc42, Rac1, and Myosin Light Chain 2 (MLC2) was analyzed using a QuantiTect SYBR Green PCR Kit (204143, QIAGEN, Hilden, Germany). β-actin mRNA was used as an internal control. qRT-PCR was run on Rotor-Gene-A (QIAGEN, Hilden, Germany) and threshold was set by 0.02 to determine the threshold cycle (Ct) value. The relative mRNA expression was calculated, as we described previously (31). Each sample was tested in duplicate, and the primer sequences used for qRT-PCR are depicted in **Table 1**.

**Table 1:** Primer sequences for quantitative real time PCR.

TAZ forward	GAGGACTTCCTCAGCAATGTGG
TAZ reverse	CGTTTGTTCCTGGAAGACAGTCA
RhoA forward	TCTGTCCCAACGTGCCCATCAT
RhoA reverse	CTGCCTTCTTCAGGTTTCACCG
RhoC forward	AAGACGAGCACACCAGGAGAGA
RhoC reverse	TTGGCTGAGCACTCAAGGTAGC
ROCK1 forward	GAAACAGTGTTCCATGCTAGACG
ROCK1 reverse	GCCGCTTATTTGATTCCTGCTCC
ROCK2 forward	TGCGGTCACAACTCCAAGCCTT
ROCK2 reverse	CGTACAGGCAATGAAAGCCATCC
Cdc42 forward	TGACAGATTACGACCGCTGAGTT
Cdc42 reverse	GGAGTCTTTGGACAGTGGTGAG
Rac1 forward	CGGTGAATCTGGGCTTATGGGA
Rac1 reverse	GGAGGTTATATCCTTACCGTACG
MLC2 forward	CGGAGAGGTTTTCCAAGGAGGA
MLC2 reverse	CTCTTCTCCGTGGGTGATGATG

### Transwell cell migration assay

Breast cancer cell lines (MCF-7 and MDA-MB-231) were transfected with 25 nM of control siRNA or TAZ-siRNA using DharmaFECT 4 reagent. After treatment with siRNA for 24 h and changing to fresh growth media, the cells were grown for another 24 h and detached using trypsin/EDTA. The detached cells were resuspended in low- or high-glucose DMEM without FBS and were seeded on the permeable filter of the transwell system (8 μm pore size, transwell) at a density of 2.0 x10^4^ cells per well. Cell migration was stimulated with DMEM media containing 10% FBS for 6 h (MDA-MB-231 cells) or for 12 h (MCF-7 cells). The upper chamber contained the medium without FBS, and the lower chamber was filled with the medium containing 10% FBS. Migrated cells on the lower face were fixed with 4% paraformaldehyde for 10 min, then stained with 0.03% crystal violet for 10 min. The numbers of the migrated cells in five randomly selected fields were counted under a light microscope at 100 X magnification.

### Scratch wound healing assay for cell migration

In 96-well IncuCyte® ImageLock Plates (Essen BioScience, Ann Arbor, MI), MDA-MB-231 and MCF7 cells were grown to confluency. Negative control-siRNA or TAZ-targeting siRNA were transfected into cells in low or high glucose DMEM when they reached 50 - 60% confluence. MDA-MB-231 and MCF-7 cells were transfected with 25 nM of either control siRNA or TAZ-siRNA. After treatment with siRNA for 24 h and changing to a fresh growth medium, cells were grown for an additional 24 h and then preincubated with mitomycin C (10 μg/mL, Sigma, St. Louis, MO) for 4 h before scratching to prevent cell proliferation. Mitomycin C treatment was maintained throughout the wound closure assay. A 96-pin WoundMaker™ (IncuCyte ZOOM® Live-Cell Imaging System; Essen BioScience, Ann Arbor, MI) was used to scratch the cell monolayer. After scratching, cell migration was induced for 24 h by 10% FBS-containing media. Every 2 h, images were automatically captured, saved, and registered by the IncuCyte™ software system. The data was analyzed using an integrated metric. The values were expressed as relative wound density.

### Fluorescence microscopy

Immunofluorescence microscopy was performed on cultured cells, as previously described (31). For immunolabeling of vinculin, cultured cells were incubated with mouse anti-vinculin monoclonal antibody (1:100, V9131, Invitrogen, Waltham, MA), followed by Alexa Fluor 488-conjugated goat anti-mouse (1:100, 16-240, sigma-aldrich). The Alexa Fluor 568 Phalloidin (A12380, ThermoFisher Scientific) dissolved in 150 μL of anhydrous DMSO to yield a 400X stock was diluted by DPBS. After incubation of the secondary antibody for vimentin immunolabeling, cells were stained with diluted phalloidin stock solution for 30 min. For double-immunolabeling of myosin light chain 2 (MLC2) and phosphorylated MLC2, cultured cells were incubated with rabbit anti-MLC2 antibody (1:100, #3672, Cell signaling Technology) and mouse anti-phosphorylated MLC2 (1:100, #3675, Cell signaling Technology), followed by Alexa Fluor 488-conjugated goat anti-rabbit (1:100, 16-240, sigma-aldrich) and Alexa Fluor 568-conjugated goat anti-mouse (1:100, sigma-aldrich). Immunofluorescence microscopy was performed using a laser scanning confocal microscope (Zeiss LSM 800; Jena, Germany).

### Next-generation sequencing

Total RNA was isolated from control-siRNA or TAZ-siRNA-treated MDA-MB-231 cells. The number of cell preparations that were included in each group was three. For transcriptome sequencing, mRNA libraries were generated using TruSeq Stranded mRNA library prep kit according to the manufacturer’s protocol (TruSeq Stranded mRNA reference guide # 1000000040498v00). Libraries were pooled and sequenced to obtain 100-bp paired-end reads on the Illumina NovaSeq platform to a depth of more than 40 million reads per sample (>10 million reads per cell). Genomic reference (GRCh38) was used to map cDNA fragments obtained through RNA-seq data. The quality of sequencing data was checked using FastQC. Trimmed reads were mapped to known reference genomes using the HISAT2 program after preprocessing. Genes/transcripts were assembled using the reference gene model through the StringTie program. After assembly, the abundance amount of the corresponding transcript was calculated using read count and normalized value; FPKM (Fragments Per Kilobase of transcript per Million mapped reads) and TPM (Transcripts Per Kilobase Million) were estimated. Gene ontology was analyzed using g:Profiler (https://biit.cs.ut.ee/gprofiler/orth) for the list of significant differentially expressed genes (DEGs). Gene set enrichment by functional classification: biological process (BP), molecular function (MF), and cellular component (CC) analyses were carried out.

### Statistical analysis

Values are presented as means ± SEM. Comparisons between the two groups were made by unpaired t-test. A comparison of multiple groups was made by one-way ANOVA followed by a *post-hoc* Bonferroni’s multiple comparison test. Multiple comparison tests were only applied when a significant difference was determined by ANOVA (*P* < 0.05). *P* values < 0.05 were considered statistically significant.

## Results

### Reduced cell migration in breast cancer cells (MCF-7 and MDA-MB-231 cells) treated with TAZ-siRNA

**Figure 1:**
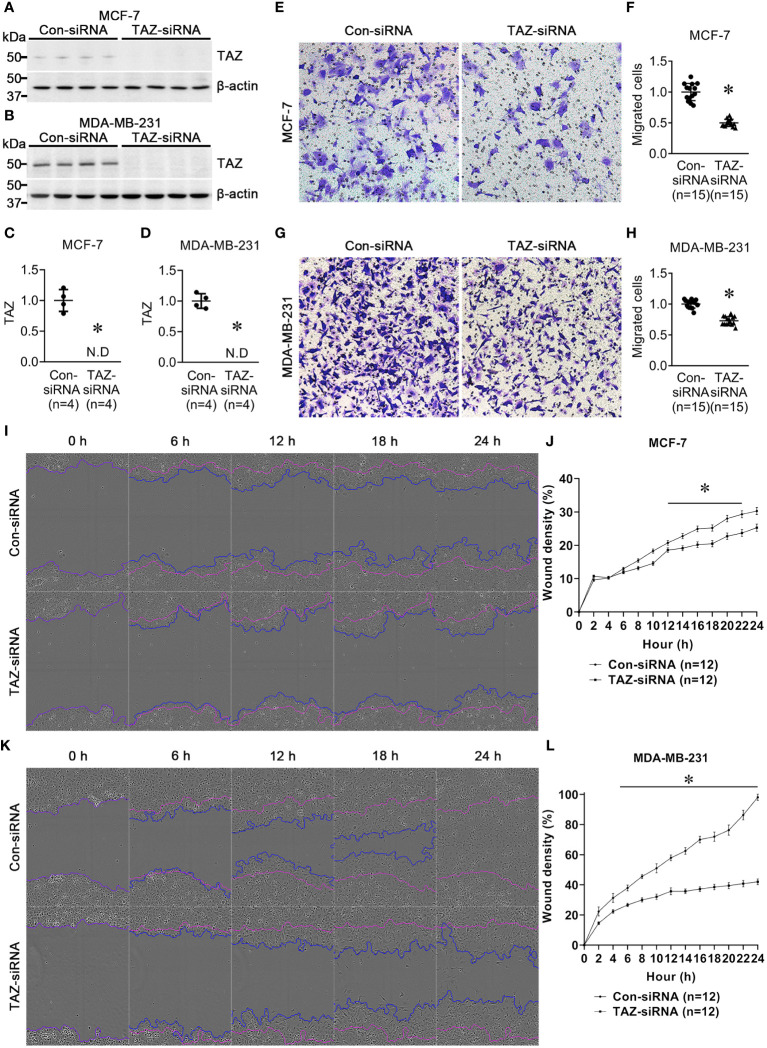
Semiquantitative immunoblotting of TAZ and cell migration assay in breast cancer cells (MCF-7 and MDA-MB-231) treated with control-siRNA or TAZ-siRNA. **(A–D)** Changes in protein abundance of TAZ in breast cancer cells treated with control-siRNA or TAZ-siRNA for 24 hours. *P < 0.05, when compared with control-siRNA. The immunoblots were reacted with antibodies against TAZ (~49 kDa) and β-actin (~42 kDa). *n* indicates the number of cell preparation in each group. **(E–H)** Transwell cell migration assay of breast cancer cells treated with control-siRNA or TAZ-siRNA. The numbers of migrated cells were counted in the randomly selected fields (x100) per well. *n* indicates the number of randomly selected fields in each group. *P < 0.05, when compared with control-siRNA. **(I–L)** Scratch wound healing assay. *P < 0.05, when compared with control-siRNA. *n* indicates the number of wells containing cell treated with control-siRNA or TAZ-siRNA.

MCF-7 and MDA-MB-231 breast cancer cell lines were treated for 24 h with control-siRNA or TAZ-siRNA. Immunoblot analysis demonstrated that TAZ knockdown was induced by TAZ-siRNA treatment in both MCF-7 and MDA-MB-231 cells (**Figures 1A–D**). We then conducted a transwell cell migration assay and a scratch wound healing assay to determine whether TAZ knockdown affects the migration of breast cancer cells. On the upper side of the transwell, serum-free DMEM media was added with cells treated with siRNA. In order to stimulate cell migration, 10% FBS was added to the DMEM media in the lower chamber. The migration of the MCF-7 cells was observed for 12 h, while MDA-MB-231 cells were observed for 6 h. TAZ knockdown significantly decreased cell migration of MCF-7 cells (49 ± 1% of control-siRNA, *P* < 0.05, **Figures 1E, F**) and MDA-MB-231 cells (73 ± 2% of control-siRNA, *P* < 0.05, **Figures 1G, H**), as determined by the transwell cell migration assay counting the numbers of migrated cells on the lower face. Furthermore, to perform the scratch wound healing assay, cells were scratched and subsequently starved for 24 h without FBS. The results demonstrated that TAZ knockdown significantly reduced cell migration in both MCF-7 and MDA-MB-231 cells (**Figures 1I–L**). Compared to control-siRNA, TAZ knockdown reduced cell migration in both MCF-7 cells (from 12 to 22 h, *P* < 0.05, **Figures 1I, J**) and MDA-MB-231 cells (from 4 to 24 h, *P* < 0.05, **Figures 1K, L**).

### Epithelial-to-mesenchymal transition in MDA-MB-231 cells with siRNA-mediated TAZ knockdown

**Figure 2:**
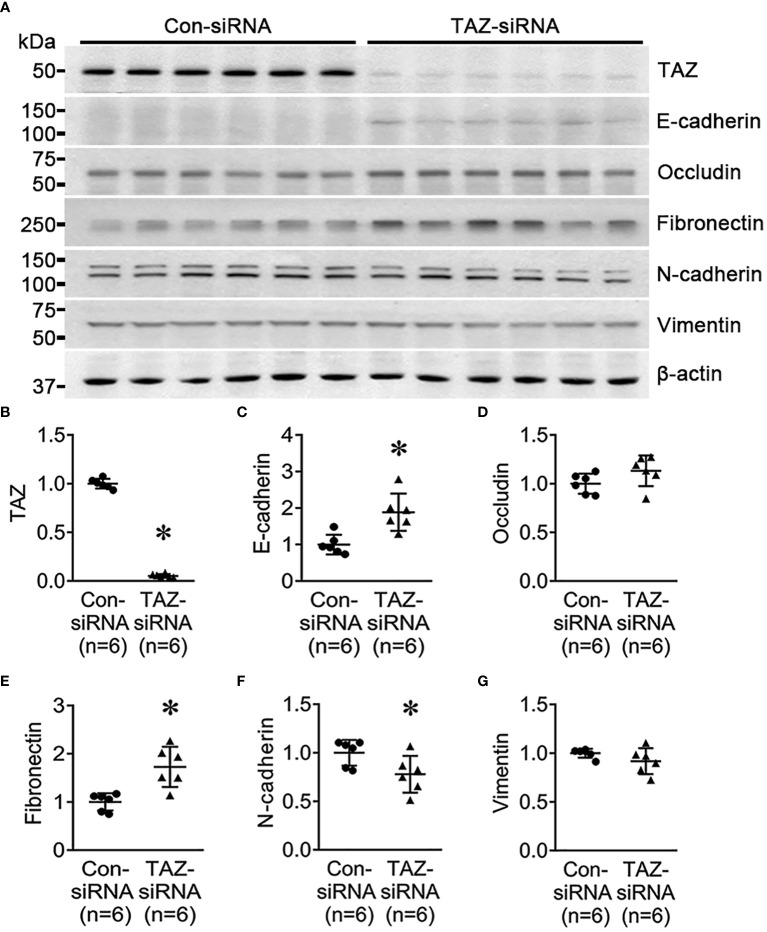
Semiquantitative immunoblotting for the studies of EMT. **(A–G)** Semiquantitative immunoblotting of TAZ, E-cadherin, occludin, fibronectin, N-cadherin, vimentin and β-actin in MDA-MB-231 cells treated with control-siRNA or TAZ-siRNA. The immunoblots were reacted with antibodies against TAZ (~49 kDa), E-cadherin (~135 kDa), occludin (~65 kDa), fibronectin (~262 kDa), N-cadherin (~125 kDa), vimentin (~57 kDa), and β-actin (~42 kDa). *P < 0.05, when compared with control-siRNA. *n* indicates the number of cell preparation in each group.

To determine if TAZ-knockdown *per se* is associated with EMT in MDA-MB-231 cells, the changes in the expression of EMT markers in breast cancer cells were examined using semiquantitative immunoblotting. As epithelial markers, E-cadherin and occludin were used, while fibronectin, N-cadherin, and vimentin were employed as mesenchymal markers. The protein abundance of E-cadherin (188 ± 23% of control-siRNA, *P* < 0.05, **Figures 2A, C**) and fibronectin (172 ± 18% of control-siRNA, *P* < 0.05, **Figures 2A, D**) was significantly increased in response to TAZ-knockdown (**Figure 2B**), whereas N-cadherin was decreased (77 ± 9% of control-siRNA, *P* < 0.05, **Figures 2A, F**). The protein levels of occludin and vimentin were unaltered (**Figures 2A, E, G**).

**Figure 3:**
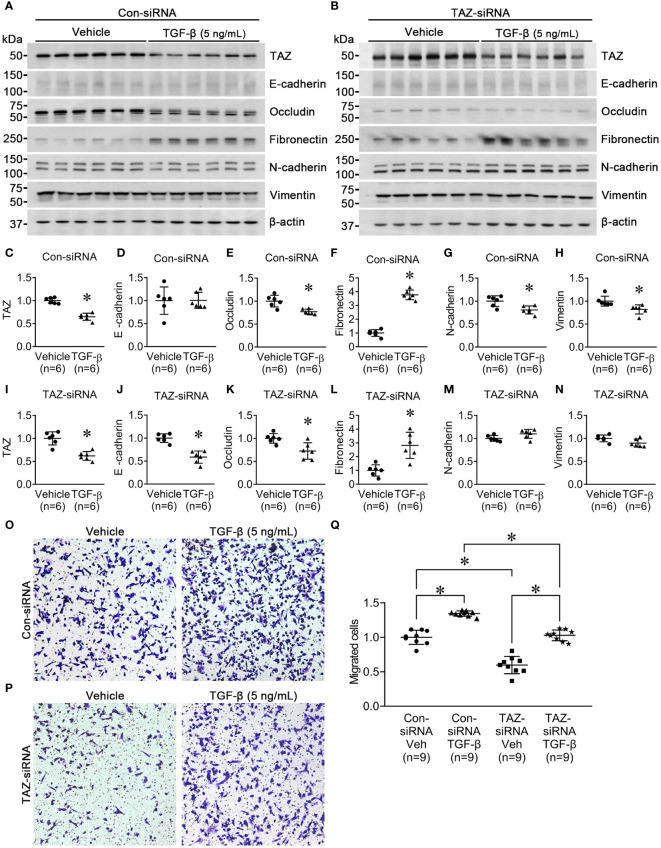
Semiquantitative immunoblotting for the studies of TGF-β treatment. **(A, C–H)** Semiquantitative immunoblotting of TAZ, E-cadherin, occludin, fibronectin, N-cadherin, vimentin, and β-actin in MDA-MB-231 cells treated with vehicle or TGF-β (5 ng/ml) under control-siRNA treatment. The immunoblots were reacted with antibodies against TAZ (~49 kDa), E-cadherin (~135 kDa), occludin (~65 kDa), fibronectin (~262 kDa), N-cadherin (~125 kDa), vimentin (~57 kDa), and β-actin (~42 kDa). **(B, I–N)** Semiquantitative immunoblotting of TAZ, E-cadherin, occludin, fibronectin, N-cadherin, vimentin, and β-actin in MDA-MB-231 cells treated with vehicle or TGF-β (5 ng/ml) under TAZ-siRNA treatment. *P < 0.05, when compared with vehicle. *n* indicates the number of cell preparation in each group. Transwell cell migration assay of MDA-MB-231 cells treated with vehicle or TGF-β (5 ng/ml) under control-siRNA **(O)** or TAZ-siRNA treatment **(P)**. **(Q)** The numbers of migrated cells were counted in the randomly selected fields (x100) per well. *n* indicates the number of randomly selected fields in each group. *P < 0.05.

Next, we investigated the effect of TGF-β (5 ng/ml, 24 h) on MDA-MB-231 cells pre-treated with control-siRNA or TAZ-siRNA. TGF-β is known to trigger EMT, cause cancer cells to develop characteristics of stem cells, and promote cell migration. We aimed to find out if TAZ knockdown in MDA-MB-231 cells could also impede TGF-β-induced cell migration and EMT. In the control-siRNA-treated MDA-MB-231 cells, TGF-β treatment significantly reduced the protein abundance of TAZ (65 ± 4% of vehicle, *P* < 0.05, **Figures 3A, C**), occludin (77 ± 5% of vehicle, *P* < 0.05, **Figures 3A, E**), N-cadherin (80 ± 6% of vehicle, *P* < 0.05, **Figures 3A, G**), and vimentin (82 ± 6% of vehicle, *P* < 0.05, **Figures 3A, H**). In contrast, TGF-β treatment increased the protein abundance of fibronectin (377 ± 14% of vehicle, *P* < 0.05, **Figures 3A, F**), but not E-cadherin (**Figures 3A, D**). In MDA-MB-231 cells with siRNA-mediated TAZ knockdown, TGF-β treatment decreased the protein abundance of TAZ further (61 ± 7% of vehicle, *P* < 0.05, **Figures 3B, I**). E-cadherin (58 ± 6% of vehicle, *P* < 0.05, **Figures 3B, J**) and occludin (72 ± 7% of vehicle, *P* < 0.05, **Figures 3B, K**) were also significantly decreased. In contrast, fibronectin protein abundance was increased (282 ± 42% of vehicle, *P* < 0.05, **Figures 3B, L**), and N-cadherin and vimentin were unchanged (**Figures 3B, M, N**) after TGF-β treatment.

Then, we examined the effects of TGF-β treatment on cell migration under TAZ knockdown, in which decreased cell migration (**Figure 1**) and increased fibronectin abundance (**Figure 2**) were observed. The transwell migration assay demonstrated that TGF-β treatment significantly increased cell migration in both control-siRNA-treated MDA-MB-231 cells (134 ± 3% of control-siRNA vehicle, *P* < 0.05, **Figures 3O, Q**) and TAZ-siRNA-treated MDA-MB-231 cells (143 ± 5% of TAZ-siRNA vehicle, *P* < 0.05, **Figures 3P, Q**).

### Next-generation sequencing in MDA-MB-231 cells with siRNA-mediated TAZ knockdown

The effects of siRNA-mediated TAZ knockdown on gene expression and gene ontology were studied using NGS. Using DESeq2, DEGs (differentially expressed genes) were identified by comparing MDA-MB-231 cells treated with control-siRNA and TAZ-siRNA. 285 genes met the criteria of fold change ≥ 2 and *P* value < 0.05. After excluding cases where expression levels were either zero or rarely detected, 140 genes were selected. **Table 2** shows the 78 genes with increased expression levels and 62 genes with decreased expression levels.

**Table 2:** Significantly changed gene expressions.

Gene_Symbol	Transcript_ID	Description	Gene expression	p-Value	*Fold change
ACAP1	NM_014716	ArfGAP with coiled-coil, ankyrin repeat and PH domains 1	upregulated	0.004949308	2.050517268
ADAM19	NM_033274	ADAM metallopeptidase domain 19	upregulated	2.26011E-34	2.241697685
ADORA1	NM_000674	adenosine A1 receptor	upregulated	7.57317E-10	2.005889332
ASB2	NM_001202429	ankyrin repeat and SOCS box containing 2	upregulated	0.004604688	2.02119655
ASPRV1	NM_152792	aspartic peptidase retroviral like 1	upregulated	0.004463118	2.36156629
ATP2B2	NM_001001331	ATPase plasma membrane Ca2+ transporting 2	upregulated	6.1251E-05	2.257761163
ATP5MF-PTCD1	NM_001198879	ATP5MF-PTCD1 readthrough	upregulated	0.01180048	2.158618851
B3GALT4	NM_003782	beta-1,3-galactosyltransferase 4	upregulated	0.002678431	2.120775696
B3GNT7	NM_145236	UDP-GlcNAc:betaGal beta-1,3-N-acetylglucosaminyltransferase 7	upregulated	0.002001943	3.695922388
BDKRB2	NM_000623	bradykinin receptor B2	upregulated	6.44346E-24	2.237463238
C1QTNF2	NM_001366504	C1q and TNF related 2	upregulated	0.001434428	2.030810691
CACNA1H	NM_001005407	calcium voltage-gated channel subunit alpha1 H	upregulated	0.000228487	2.29453092
CALHM5	NM_153711	calcium homeostasis modulator family member 5	upregulated	1.21204E-05	2.585085982
CCDC146	NM_020879	coiled-coil domain containing 146	upregulated	1.33824E-08	2.247317851
CNIH3	NM_001322302	cornichon family AMPA receptor auxiliary protein 3	upregulated	2.76126E-15	2.04075093
CREB3L3	NM_001271995	cAMP responsive element binding protein 3 like 3	upregulated	0.000785913	2.141721197
CST1	NM_001898	cystatin SN	upregulated	2.29545E-23	2.126122181
CST2	NM_001322	cystatin SA	upregulated	1.79636E-08	2.077961636
CTTNBP2	NM_001363349	cortactin binding protein 2	upregulated	0.002054734	2.065075445
CYGB	NM_134268	cytoglobin	upregulated	3.01827E-05	2.408874975
EBF2	NM_022659	EBF transcription factor 2	upregulated	6.03458E-05	3.352858635
EGR2	NM_000399	early growth response 2	upregulated	2.48677E-28	2.10662509
EPHB1	NM_004441	EPH receptor B1	upregulated	0.000315418	2.10017562
FRMPD3	NM_032428	FERM and PDZ domain containing 3	upregulated	9.90805E-05	2.057414227
GALNT9	NM_001122636	polypeptide N-acetylgalactosaminyltransferase 9	upregulated	0.000471314	2.153385179
GAS7	NM_001130831	growth arrest specific 7	upregulated	5.99582E-11	2.530431268
GLI1	NM_001160045	GLI family zinc finger 1	upregulated	0.000975769	2.386161697
GLI2	NM_001371271	GLI family zinc finger 2	upregulated	3.08373E-09	2.112878586
GPR75-ASB3	NM_001164165	GPR75-ASB3 readthrough	upregulated	0.000433613	10.14243653
GRIK4	NM_001282470	glutamate ionotropic receptor kainate type subunit 4	upregulated	0.005584754	2.280123644
GRIN2D	NM_000836	glutamate ionotropic receptor NMDA type subunit 2D	upregulated	0.003465746	2.212926712
HOXC4	NM_014620	homeobox C4	upregulated	0.002403674	2.570203546
HS3ST5	NM_153612	heparan sulfate-glucosamine 3-sulfotransferase 5	upregulated	0.001772065	2.987878292
ISY1-RAB43	NM_001204890	ISY1-RAB43 readthrough	upregulated	0.002365041	2.091108351
ITGB8	NM_002214	integrin subunit beta 8	upregulated	2.57859E-10	2.879197568
JAKMIP2	NM_001270934	janus kinase and microtubule interacting protein 2	upregulated	4.40569E-15	2.289780135
KIAA0319	NM_001168374	KIAA0319	upregulated	3.50748E-05	2.036202765
LINGO2	NM_001258282	leucine rich repeat and Ig domain containing 2	upregulated	0.000259012	2.100330628
LRP1B	NM_018557	LDL receptor related protein 1B	upregulated	3.08927E-11	2.196172524
LYNX1	NM_001356370	Ly6/neurotoxin 1	upregulated	5.58439E-06	2.261644166
MAP1A	NM_002373	microtubule associated protein 1A	upregulated	5.65799E-07	2.042410989
MEF2B	NM_001145785	myocyte enhancer factor 2B	upregulated	0.007041908	3.222022292
MMP13	NM_002427	matrix metallopeptidase 13	upregulated	1.69723E-05	2.189041085
MUC5AC	NM_001304359	mucin 5AC, oligomeric mucus/gel-forming	upregulated	6.96806E-07	2.335975675
NCALD	NM_001040624	neurocalcin delta	upregulated	7.39606E-06	2.047862034
NEURL1B	NM_001142651	neuralized E3 ubiquitin protein ligase 1B	upregulated	1.46035E-15	2.084240965
NOS2	NM_000625	nitric oxide synthase 2	upregulated	0.006064819	2.917128143
NTNG2	NM_032536	netrin G2	upregulated	1.10923E-18	2.647092514
PAPPA	NM_002581	pappalysin 1	upregulated	6.50121E-05	2.076060673
PCDHGA10	NM_018913	protocadherin gamma subfamily A, 10	upregulated	2.17784E-11	2.184171382
PCDHGA4	NM_018917	protocadherin gamma subfamily A, 4	upregulated	0.001059173	2.867036292
PCDHGB2	NM_018923	protocadherin gamma subfamily B, 2	upregulated	9.60892E-05	2.067449003
PDE3A	NM_000921	phosphodiesterase 3A	upregulated	2.95899E-06	2.546710938
PIWIL4	NM_152431	piwi like RNA-mediated gene silencing 4	upregulated	0.001863325	2.558595344
PLA2G3	NM_015715	phospholipase A2 group III	upregulated	0.009640603	2.523948258
PRSS2	NM_001303414	serine protease 2	upregulated	1.01379E-06	4.049252026
RAPGEF4	NM_001100397	Rap guanine nucleotide exchange factor 4	upregulated	1.87693E-08	2.09450615
RIBC1	NM_001031745	RIB43A domain with coiled-coils 1	upregulated	0.006111328	2.352863559
RSAD2	NM_080657	radical S-adenosyl methionine domain containing 2	upregulated	1.79674E-10	2.686175673
SCN3A	NM_001081676	sodium voltage-gated channel alpha subunit 3	upregulated	1.96522E-18	2.339632917
SDC3	NM_014654	syndecan 3	upregulated	3.30358E-23	2.129624098
SEMA6B	NM_032108	semaphorin 6B	upregulated	3.32935E-19	2.033166468
SHC3	NM_016848	SHC adaptor protein 3	upregulated	2.39484E-16	2.059833666
SHISAL1	NM_001099294	shisa like 1	upregulated	1.59111E-43	4.371413572
SIDT1	NM_001308350	SID1 transmembrane family member 1	upregulated	5.19619E-08	2.184384332
SKOR1	NM_001365915	SKI family transcriptional corepressor 1	upregulated	5.52554E-05	2.605223616
SLC16A14	NM_152527	solute carrier family 16 member 14	upregulated	8.96874E-08	2.805860918
SLC7A9	NM_001126335	solute carrier family 7 member 9	upregulated	0.008918305	2.234025665
SLCO3A1	NM_001145044	solute carrier organic anion transporter family member 3A1	upregulated	3.77828E-28	2.029622149
SLIT1	NM_003061	slit guidance ligand 1	upregulated	0.000330772	2.601772769
SNED1	NM_001080437	sushi, nidogen and EGF like domains 1	upregulated	5.38365E-27	2.063355178
SORCS2	NM_020777	sortilin related VPS10 domain containing receptor 2	upregulated	0.004475073	2.793598438
SOX5	NM_001261414	SRY-box transcription factor 5	upregulated	3.32301E-09	2.299998702
SPOCK3	NM_001040159	SPARC (osteonectin), cwcv and kazal like domains proteoglycan 3	upregulated	2.61619E-13	2.153042128
STON1-GTF2A1L	NM_001198593	STON1-GTF2A1L readthrough	upregulated	0.011749062	2.527554438
TRPC3	NM_001130698	transient receptor potential cation channel subfamily C member 3	upregulated	7.37691E-06	2.003098213
TSPAN2	NM_001308315	tetraspanin 2	upregulated	8.34757E-23	2.57367749
WNT2B	NM_001291880	Wnt family member 2B	upregulated	1.11759E-10	2.014696062
ADAMTS6	NM_197941	ADAM metallopeptidase with thrombospondin type 1 motif 6	downregulated	1.33754E-18	-2.947030193
ADORA2A	NM_000675	adenosine A2a receptor	downregulated	0.000138245	-5.610140894
ADRB2	NM_000024	adrenoceptor beta 2	downregulated	5.46856E-30	-2.165878349
ALPP	NM_001632	alkaline phosphatase, placental	downregulated	2.95397E-07	-4.92898576
ANKRD1	NM_014391	ankyrin repeat domain 1	downregulated	1.0413E-239	-7.642980386
ANXA8	NM_001040084	annexin A8	downregulated	1.05811E-09	-2.045346899
ATP6V0A4	NM_020632	ATPase H+ transporting V0 subunit a4	downregulated	1.23259E-20	-2.867355966
BHLHA15	NM_177455	basic helix-loop-helix family member a15	downregulated	3.61056E-06	-2.036769741
BIRC3	NM_001165	baculoviral IAP repeat containing 3	downregulated	3.77772E-23	-2.03557939
CALCB	NM_000728	calcitonin related polypeptide beta	downregulated	0.006008249	-2.006325926
CAVIN2	NM_004657	caveolae associated protein 2	downregulated	1.95421E-37	-2.508809933
CCBE1	NM_133459	collagen and calcium binding EGF domains 1	downregulated	5.94893E-12	-2.01164236
CCDC187	NM_001291516	coiled-coil domain containing 187	downregulated	0.008829489	-2.474151393
CCN1	NM_001554	cellular communication network factor 1	downregulated	8.95353E-47	-2.682098404
CCN2	NM_001901	cellular communication network factor 2	downregulated	2.58103E-36	-2.023159559
CD33	NM_001082618	CD33 molecule	downregulated	0.00355832	-2.152520404
CLDN1	NM_021101	claudin 1	downregulated	5.44775E-64	-3.99767905
CYB5R2	NM_001302826	cytochrome b5 reductase 2	downregulated	0.002805243	-2.314005407
DKK1	NM_012242	dickkopf WNT signaling pathway inhibitor 1	downregulated	8.6222E-115	-3.421346763
DMBT1	NM_001320644	deleted in malignant brain tumors 1	downregulated	6.18097E-19	-2.420670798
DNAH10	NM_001372106	dynein axonemal heavy chain 10	downregulated	0.000264421	-2.241281661
EDN1	NM_001168319	endothelin 1	downregulated	1.22556E-21	-2.610701767
FAM71D	NM_173526	family with sequence similarity 71 member D	downregulated	2.09536E-07	-4.874890615
FCMR	NM_001142473	Fc fragment of IgM receptor	downregulated	8.29639E-05	-2.723808531
FGF1	NM_000800	fibroblast growth factor 1	downregulated	7.87286E-05	-2.957285531
FSBP	NM_001256141	fibrinogen silencer binding protein	downregulated	0.00499564	-4.225295114
IGF2	NM_000612	insulin like growth factor 2	downregulated	0.001368233	-2.935129821
IL32	NM_001012631	interleukin 32	downregulated	2.6262E-08	-2.229805348
INSL4	NM_002195	insulin like 4	downregulated	0.003824492	-2.351407347
KISS1	NM_002256	KiSS-1 metastasis suppressor	downregulated	3.59686E-15	-2.813016872
KLF15	NM_014079	Kruppel like factor 15	downregulated	4.04905E-09	-2.624248806
KLHDC7B	NM_138433	kelch domain containing 7B	downregulated	0.000643973	-2.74562398
KRT34	NM_021013	keratin 34	downregulated	2.99731E-06	-2.438219034
KRTAP2-3	NM_001165252	keratin associated protein 2-3	downregulated	1.82545E-70	-28.21070197
LAMA1	NM_005559	laminin subunit alpha 1	downregulated	1.6628E-08	-2.277195707
LINC01638	NM_001350812	long intergenic non-protein coding RNA 1638	downregulated	0.012699043	-2.051900622
LPAR1	NM_001351397	lysophosphatidic acid receptor 1	downregulated	1.98376E-56	-3.563934724
MIOX	NM_017584	myo-inositol oxygenase	downregulated	1.81877E-07	-3.236151315
MYPN	NM_001256267	myopalladin	downregulated	1.19265E-06	-2.918838385
NCKAP1L	NM_001184976	NCK associated protein 1 like	downregulated	1.56938E-05	-2.685570058
NGF	NM_002506	nerve growth factor	downregulated	1.15007E-05	-2.482904593
NNMT	NM_001372045	nicotinamide N-methyltransferase	downregulated	0.00010588	-2.451627125
NUPR1	NM_001042483	nuclear protein 1, transcriptional regulator	downregulated	2.36919E-08	-2.304730661
OLAH	NM_001039702	oleoyl-ACP hydrolase	downregulated	2.82214E-05	-4.434731739
PAWR	NM_001354732	pro-apoptotic WT1 regulator	downregulated	2.16924E-58	-2.009112218
PDGFB	NM_002608	platelet derived growth factor subunit B	downregulated	7.0234E-20	-2.500457341
PIK3R5	NM_001142633	phosphoinositide-3-kinase regulatory subunit 5	downregulated	0.002895495	-2.979348849
PRR16	NM_001300783	proline rich 16	downregulated	5.34033E-10	-2.019548481
RELN	NM_005045	reelin	downregulated	0.000737683	-2.39185058
RGS7	NM_001282773	regulator of G protein signaling 7	downregulated	0.004049836	-2.069475563
RIMS2	NM_001100117	regulating synaptic membrane exocytosis 2	downregulated	1.00694E-20	-3.412280862
SCEL	NM_001160706	sciellin	downregulated	9.95617E-11	-5.055784635
SNAPC1	NM_003082	small nuclear RNA activating complex polypeptide 1	downregulated	1.09615E-68	-2.102240661
ST6GALNAC5	NM_001320273	ST6 N-acetylgalactosaminide alpha-2,6-sialyltransferase 5	downregulated	5.3001E-13	-5.875676597
STK31	NM_001260504	serine/threonine kinase 31	downregulated	1.11305E-12	-3.184050246
STMN3	NM_001276310	stathmin 3	downregulated	0.000998287	-2.503628441
TGFB2	NM_001135599	transforming growth factor beta 2	downregulated	1.9667E-55	-2.544268019
TNFSF15	NM_001204344	TNF superfamily member 15	downregulated	0.000385755	-2.409755599
TNFSF18	NM_005092	TNF superfamily member 18	downregulated	9.64041E-05	-3.788137775
TREX1	NM_007248	three prime repair exonuclease 1	downregulated	3.83515E-05	-2.162873135
WWTR1	NM_001168278	WW domain containing transcription regulator 1	downregulated	0	-11.55672309

*Fold change: siRNA-mediated TAZ knockdown vs. control-siRNA treatment in MDA-MB-231 cells.

Next, NGS data were categorized using the Pathcards database (https://pathcards.genecards.org), an integrated database consolidating 1,626 SuperPath entries from 11 sources and clustering human pathways based on gene content similarity. Each PathCard offers information about a SuperPath, representing one or more human pathways (32). The superpathway regarding the “cytoskeleton remodeling regulation of actin cytoskeleton by Rho GTPases” had the highest relevance score among the actin-related pathways (https://pathcards.genecards.org/Search/Results?query=actin). The superpathway includes 5 subpathways. A total of 187 genes were included in the superpathways database, and 68 genes from our NGS data were found in the superpathways (**Table 3**). The superpathway regarding the “actin nucleation by ARP-WASP complex” had the second highest relevance score among the actin-related pathways. The superpathway consists of 4 subpathways, including a total of 344 genes, and 109 genes in our NGS data were found in the superpathways (**Table 4**).

**Table 3:** Cytoskeleton remodeling regulation of actin cytoskeleton by Rho GTPases.

Gene symbol	Description	*Fold change	p-Value	Pathways				
				1	2	3	4	5
CLDN1	claudin 1	-3.99767905	5.4477E-64	o	x	o	x	o
NGF	nerve growth factor	-2.482904593	1.1501E-05	x	x	x	o	x
MYH16	myosin heavy chain 16 pseudogene	-2.456489246	0.02661264	x	o	o	x	x
LAMA1	laminin subunit alpha 1	-2.277195707	1.6628E-08	x	x	x	o	x
MYLK	myosin light chain kinase	-1.766201798	9.2088E-18	x	x	o	x	x
CAV1	caveolin 1	-1.517664331	2.8082E-31	o	x	o	x	x
TJP3	tight junction protein 3	-1.498195657	0.02635195	o	o	x	x	x
BDNF	brain derived neurotrophic factor	-1.35723822	5.6757E-10	x	x	x	o	x
LIMK1	LIM domain kinase 1	-1.283294164	1.356E-09	o	x	o	x	o
MYL5	myosin light chain 5	-1.24055366	0.0363567	o	x	x	x	o
MTDH	metadherin	-1.195451363	6.4973E-05	x	x	x	x	o
PARVA	parvin alpha	-1.147989421	0.00068592	x	o	x	o	x
ARPC1A	actin related protein 2/3 complex subunit 1A	-1.142329517	0.00121682	x	x	x	o	x
ACTG1	actin gamma 1	-1.139588554	0.00126064	x	x	o	o	x
LAMB1	laminin subunit beta 1	-1.137453523	0.00476849	o	x	o	o	o
ARPC3	actin related protein 2/3 complex subunit 3	-1.134523376	0.00310162	o	o	x	x	x
AKT1	AKT serine/threonine kinase 1	-1.122800151	0.00377361	o	o	x	x	x
ACTR2	actin related protein 2	-1.113349939	0.02293064	x	x	x	x	o
PFN1	profilin 1	-1.102688536	0.04932765	x	o	x	x	x
MAP2K2	mitogen-activated protein kinase kinase 2	-1.101010313	0.02770762	x	o	x	x	x
LIMS1	LIM zinc finger domain containing 1	-1.099780659	0.02882081	o	o	x	x	x
TJP1	tight junction protein 1	-1.096777222	0.03364182	o	o	x	x	x
RAC1	Rac family small GTPase 1	-1.095428061	0.02060815	x	x	x	o	x
RHOA	ras homolog family member A	-1.081022243	0.04842445	x	o	x	x	o
NECTIN1	nectin cell adhesion molecule 1	1.822283021	1.6983E-11	x	x	x	x	o
CGN	cingulin	1.519925052	0.00020374	o	x	o	x	o
ACTA2	actin alpha 2, smooth muscle	1.474911523	0.00037032	x	x	x	o	x
ITGA5	integrin subunit alpha 5	1.42891795	6.2022E-19	x	o	x	x	x
ITGA2	integrin subunit alpha 2	1.410654554	2.8573E-17	x	x	x	o	x
COL1A1	collagen type I alpha 1 chain	1.341339411	8.1901E-05	o	o	o	x	x
CLDN4	claudin 4	1.332312801	1.0855E-05	x	x	x	x	o
F11R	F11 receptor	1.329528093	2.8868E-10	o	x	o	o	o
PIP5K1C	phosphatidylinositol-4-phosphate 5-kinase type 1 gamma	1.323904977	8.9421E-06	x	x	x	x	o
LAMC1	laminin subunit gamma 1	1.310431122	5.196E-09	x	x	o	x	x
SOS1	SOS Ras/Rac guanine nucleotide exchange factor 1	1.309673303	3.1039E-08	x	x	x	o	x
GSK3B	glycogen synthase kinase 3 beta	1.284382287	1.6098E-05	o	x	o	o	o
COL4A2	collagen type IV alpha 2 chain	1.284017641	1.5696E-06	o	o	o	x	x
NKX2-1	NK2 homeobox 1	1.224347264	0.00039888	o	o	o	x	x
TCF7L1	transcription factor 7 like 1	1.214999894	0.03303257	x	x	x	x	o
ITGA3	integrin subunit alpha 3	1.209847644	6.5632E-06	x	o	x	x	x
VCL	vinculin	1.202821163	2.8119E-05	o	o	o	x	x
ARHGDIA	Rho GDP dissociation inhibitor alpha	1.200288011	1.5837E-05	x	x	x	x	o
JUN	Jun proto-oncogene, AP-1 transcription factor subunit	1.189242711	0.00313305	x	x	x	o	x
MYL9	myosin light chain 9	1.187975048	3.1899E-05	o	o	x	x	x
ITGB3	integrin subunit beta 3	1.186807455	0.02016037	x	o	x	x	x
AKT3	AKT serine/threonine kinase 3	1.179099523	0.00061083	x	x	x	x	o
AFDN	afadin, adherens junction formation factor	1.170414867	0.00180193	o	x	x	x	x
PAK1	p21 (RAC1) activated kinase 1	1.163451028	0.0006292	o	x	o	o	o
OCLN	occludin	1.163300921	0.01288947	x	o	x	x	x
FSCN1	fascin actin-bundling protein 1	1.154980882	0.00047982	x	o	x	x	x
PSEN1	presenilin 1	1.153257277	0.00073728	o	o	x	x	x
ITGAV	integrin subunit alpha V	1.1530368	0.00185572	x	x	x	o	x
COL4A1	collagen type IV alpha 1 chain	1.15161724	0.01119491	x	o	x	x	x
MYH10	myosin heavy chain 10	1.14590334	0.02421947	o	x	o	o	o
CFL2	cofilin 2	1.145469135	0.00048494	x	o	x	x	x
APH1B	aph-1 homolog B, gamma-secretase subunit	1.137905039	0.04616204	o	o	x	x	x
MPDZ	multiple PDZ domain crumbs cell polarity complex component	1.133446898	0.02275105	o	x	o	x	x
PTK2	protein tyrosine kinase 2	1.119547762	0.0108876	o	o	x	x	x
DOCK1	dedicator of cytokinesis 1	1.113358015	0.04466996	x	x	x	o	x
FN1	fibronectin 1	1.110664249	0.00607455	o	o	o	x	x
MAPK1	mitogen-activated protein kinase 1	1.110169679	0.01551715	o	o	x	x	x
ITGB1	integrin subunit beta 1	1.104527597	0.00940893	o	o	o	x	x
CTNNB1	catenin beta 1	1.100673225	0.00977357	o	x	x	x	x
ARHGAP32	Rho GTPase activating protein 32	1.099262279	0.03856255	o	x	o	x	x
CDC42	cell division cycle 42	1.096140069	0.01942584	o	o	x	x	x
PPP1CB	protein phosphatase 1 catalytic subunit beta	1.090758523	0.0204361	x	x	x	o	x
ARPC5	actin related protein 2/3 complex subunit 5	1.088349017	0.02903029	x	x	x	o	x
ACTB	actin beta	1.085123631	0.03165157	o	o	o	o	o

*Fold change: siRNA-mediated TAZ knockdown vs. control-siRNA treatment in MDA-MB-231 cells.

Five pathways included in the Cytoskeleton remodeling Regulation of actin cytoskeleton by Rho GTPases.

1. Cell adhesion Integrin-mediated cell adhesion and migration.

2. Cytoskeleton remodeling Integrin outside-in signaling.

3. Cytoskeleton remodeling Regulation of actin cytoskeleton by Rho GTPases.

4. Cell adhesion Tight junctions.

5. Development MAG-dependent inhibition of neurite outgrowth.

o indicates the genes involved in each pathway.

x indicates the genes not involved in each pathway.

**Table 4:** Actin nucleation by ARP-WASP complex.

Gene symbol	Description	*Fold change	p-Value	Pathways			
				1	2	3	4
LPAR1	lysophosphatidic acid receptor 1	-3.563934724	1.9838E-56	o	x	x	x
IGF2	insulin like growth factor 2	-2.935129821	0.00136823	o	x	x	x
MYLK	myosin light chain kinase	-1.766201798	9.2088E-18	o	x	x	o
ARHGEF16	Rho guanine nucleotide exchange factor 16	-1.341379419	0.00116608	o	x	x	x
DIAPH3	diaphanous related formin 3	-1.322755836	5.2985E-09	x	x	x	o
ANAPC11	anaphase promoting complex subunit 11	-1.311004082	3.3428E-05	x	x	x	o
FGFR4	fibroblast growth factor receptor 4	-1.304664841	0.04253518	x	o	o	o
LIMK1	LIM domain kinase 1	-1.283294164	1.356E-09	o	x	o	o
AXL	AXL receptor tyrosine kinase	-1.272280842	6.9603E-09	x	o	o	o
CDC42EP2	CDC42 effector protein 2	-1.253520617	0.00016956	o	x	x	o
MYL5	myosin light chain 5	-1.24055366	0.0363567	o	o	x	x
MYO1C	myosin IC	-1.240502602	6.912E-09	o	o	x	x
CDC42EP1	CDC42 effector protein 1	-1.223862456	1.6408E-05	o	x	x	x
ARHGAP1	Rho GTPase activating protein 1	-1.191913598	0.00013602	o	x	x	o
RHOD	ras homolog family member D	-1.186898355	0.02239112	x	o	o	x
CDC16	cell division cycle 16	-1.164967396	0.00022158	x	x	x	o
PKN1	protein kinase N1	-1.162509401	4.7476E-05	o	x	x	x
EGFR	epidermal growth factor receptor	-1.154558601	0.00039365	x	o	o	o
ANAPC13	anaphase promoting complex subunit 13	-1.154069336	0.00442327	x	x	x	o
RHOQ	ras homolog family member Q	-1.151634797	0.02200722	x	o	o	x
MET	MET proto-oncogene, receptor tyrosine kinase	-1.145460301	0.00508763	x	o	o	o
ACTG1	actin gamma 1	-1.139588554	0.00126064	o	o	o	o
SLC9A1	solute carrier family 9 member A1	-1.138542976	0.04042663	o	x	x	x
NCK1	NCK adaptor protein 1	-1.135705274	0.01043462	x	o	x	x
EXOC1	exocyst complex component 1	-1.128054763	0.00435309	x	x	x	o
EXOC4	exocyst complex component 4	-1.116996551	0.02846444	x	x	x	o
ARHGEF1	Rho guanine nucleotide exchange factor 1	-1.11474951	0.01183559	o	x	x	x
ANAPC1	anaphase promoting complex subunit 1	-1.113967328	0.04915229	x	x	x	o
ACTR2	actin related protein 2	-1.113349939	0.02293064	o	o	o	o
MYO1B	myosin IB	-1.112310896	0.00713856	o	o	x	x
PFN1	profilin 1	-1.102688536	0.04932765	o	x	o	x
RAC1	Rac family small GTPase 1	-1.095428061	0.02060815	x	o	o	x
ANAPC5	anaphase promoting complex subunit 5	-1.093957201	0.04915229	x	x	x	o
RAC2	Rac family small GTPase 2	-1.090616496	0.04780764	x	o	o	x
RHOA	ras homolog family member A	-1.081022243	0.04842445	o	o	o	x
SEPTIN2	septin 2	-1.077901985	0.03785428	o	x	3x	x
ITGB8	integrin subunit beta 8	2.879197568	2.5786E-10	o	o	o	o
EPHB1	EPH receptor B1	2.10017562	0.00031542	x	o	o	o
PAK6	p21 (RAC1) activated kinase 6	1.949324406	0.00543178	x	x	o	x
LMTK3	lemur tyrosine kinase 3	1.745155208	0.0244554	x	o	o	o
RHOB	ras homolog family member B	1.689607967	6.6832E-21	x	o	o	x
IQGAP2	IQ motif containing GTPase activating protein 2	1.671468429	4.3329E-11	x	x	x	o
PTK2B	protein tyrosine kinase 2 beta	1.655828278	4.0174E-08	o	x	x	x
ARHGEF4	Rho guanine nucleotide exchange factor 4	1.641362862	8.1958E-08	o	x	x	x
MCF2L	MCF.2 cell line derived transforming sequence like	1.50313654	0.00013788	o	x	x	x
ACTA2	actin alpha 2, smooth muscle	1.474911523	0.00037032	o	o	o	o
NET1	neuroepithelial cell transforming 1	1.438739422	6.8135E-14	o	x	x	x
ITGA5	integrin subunit alpha 5	1.42891795	6.2022E-19	o	o	o	o
DDR1	discoidin domain receptor tyrosine kinase 1	1.425857275	5.8597E-11	x	o	o	o
ITGA2	integrin subunit alpha 2	1.410654554	2.8573E-17	o	o	o	o
FGFR1	fibroblast growth factor receptor 1	1.385355239	3.0876E-16	x	o	o	o
MAP3K12	mitogen-activated protein kinase kinase kinase 12	1.378139035	2.3852E-06	x	x	x	o
SEPTIN4	septin 4	1.372521899	0.00611883	o	x	x	x
CHN2	chimerin 2	1.369051099	0.00047132	o	x	x	x
PDGFRB	platelet derived growth factor receptor beta	1.354335269	4.5742E-06	x	o	o	o
LIFR	LIF receptor subunit alpha	1.351314061	1.1386E-07	x	o	o	o
PIP4K2B	phosphatidylinositol-5-phosphate 4-kinase type 2 beta	1.336482992	2.9871E-07	o	x	x	x
PIP5K1C	phosphatidylinositol-4-phosphate 5-kinase type 1 gamma	1.323904977	8.9421E-06	o	x	x	x
ITGA6	integrin subunit alpha 6	1.322962717	8.6525E-08	o	o	o	o
EPHB3	EPH receptor B3	1.320573823	0.00429305	x	o	o	o
GNAZ	G protein subunit alpha z	1.308554099	0.01242951	x	o	o	x
MYO1E	myosin IE	1.295857547	1.1979E-07	o	o	x	x
ARHGEF3	Rho guanine nucleotide exchange factor 3	1.295099694	1.8085E-05	o	x	x	x
ITGB5	integrin subunit beta 5	1.294068834	2.6966E-08	o	o	o	o
FGD1	FYVE, RhoGEF and PH domain containing 1	1.293390365	0.00276582	x	x	x	o
CHN1	chimerin 1	1.292968441	8.408E-07	o	x	x	x
GSK3B	glycogen synthase kinase 3 beta	1.284382287	1.6098E-05	x	x	x	o
ITGB4	integrin subunit beta 4	1.264871913	2.0347E-05	o	o	o	o
ARHGAP5	Rho GTPase activating protein 5	1.255481781	6.8416E-06	o	x	x	x
HLA-C	major histocompatibility complex, class I, C	1.254438349	1.2528E-06	x	x	x	o
HLA-A	major histocompatibility complex, class I, A	1.246355082	8.0578E-07	x	x	x	o
EPHB4	EPH receptor B4	1.243643416	0.00118085	x	o	o	o
HLA-F	major histocompatibility complex, class I, F	1.238993345	0.04650243	x	x	x	o
EPHB2	EPH receptor B2	1.224566405	0.00068172	x	o	o	o
HLA-B	major histocompatibility complex, class I, B	1.215249703	0.00014349	x	x	x	o
ITGA3	integrin subunit alpha 3	1.209847644	6.5632E-06	o	o	o	o
VCL	vinculin	1.202821163	2.8119E-05	o	x	x	x
ARHGDIA	Rho GDP dissociation inhibitor alpha	1.200288011	1.5837E-05	o	o	x	x
IGF1R	insulin like growth factor 1 receptor	1.199220562	0.01572043	o	o	o	o
JUN	Jun proto-oncogene, AP-1 transcription factor subunit	1.189242711	0.00313305	x	x	x	o
MYL9	myosin light chain 9	1.187975048	3.1899E-05	o	o	x	o
MYO9B	myosin IXB	1.187943016	0.00428323	o	o	x	x
PLD1	phospholipase D1	1.187738026	0.04987579	o	x	x	x
EPHA2	EPH receptor A2	1.187549756	0.00034545	x	o	o	o
ITGB3	integrin subunit beta 3	1.186807455	0.02016037	o	o	o	o
MYO5A	myosin VA	1.186442248	0.00036971	o	o	x	x
PPP1R12B	protein phosphatase 1 regulatory subunit 12B	1.184630095	0.04034509	o	o	x	o
ERBB2	erb-b2 receptor tyrosine kinase 2	1.183609587	0.01206792	x	o	o	o
EXOC2	exocyst complex component 2	1.180644057	0.00091177	x	x	x	o
INSR	insulin receptor	1.178764715	0.0388677	x	o	o	o
MYO10	myosin X	1.175458354	0.00141153	o	o	x	x
LMTK2	lemur tyrosine kinase 2	1.167684492	0.0038115	x	o	o	o
PTK7	protein tyrosine kinase 7 (inactive)	1.164691586	0.00475138	x	o	o	o
PAK1	p21 (RAC1) activated kinase 1	1.163451028	0.0006292	x	x	o	o
ITGAV	integrin subunit alpha V	1.1530368	0.00185572	o	o	o	o
ARHGEF10	Rho guanine nucleotide exchange factor 10	1.148558901	0.02088124	o	x	x	x
MYH10	myosin heavy chain 10	1.14590334	0.02421947	o	o	x	x
CFL2	cofilin 2	1.145469135	0.00048494	o	x	o	o
GNAI1	G protein subunit alpha i1	1.135917237	0.01154773	o	o	o	x
CDC23	cell division cycle 23	1.133939819	0.00520006	x	x	x	o
GNB4	G protein subunit beta 4	1.129748179	0.03846163	x	o	o	x
ARHGEF11	Rho guanine nucleotide exchange factor 11	1.127955696	0.04200375	o	x	x	x
GNB5	G protein subunit beta 5	1.125092329	0.00858585	x	o	o	x
PTK2	protein tyrosine kinase 2	1.119547762	0.0108876	o	x	x	x
FNBP1L	formin binding protein 1 like	1.116873793	0.01280916	x	x	x	o
CDC42EP4	CDC42 effector protein 4	1.116132825	0.01300773	o	x	x	x
ITGB1	integrin subunit beta 1	1.104527597	0.00940893	o	o	o	o
CDC42	cell division cycle 42	1.096140069	0.01942584	x	o	o	o
ACTB	actin beta	1.085123631	0.01735541	o	o	o	o

*Fold change: siRNA-mediated TAZ knockdown vs. control-siRNA treatment in MDA-MB-231 cells.

Four pathways included in Actin nucelation by ARP-WASP complex.

1. RhoA pathway.

2. Actin nucleation by ARP-WASP complex.

3. Actin nucleation and Branching.

4. CDC42 pathway.

o indicates the genes involved in each pathway.

x indicates the genes not involved in each pathway.

In **Tables 3**, **4** demonstrating NGS data, MYLK, LIMK1, and LPAR1, which are involved in RhoA/ROCK-mediated actin dynamics, were downregulated in response to TAZ knockdown. Moreover, several genes which are involved in regulation of actin dynamics and Rho GTPases, such as *ACTG1, ARPC2, ACTR2, RAC1, RHOA, ARHGEF16, DIAPH3, ARHGAP1, RHOD, RHOQ, ARHGEF1, PFN1*, and *RAC2*, were also downregulated in MDA-MB-231 cells with TAZ knockdown.

Next, g:Profiler (https://biit.cs.ut.ee/gprofiler/orth) was used to examine gene ontology to compile a list of important DEGs. Gene set enrichment using functional classification, e.g., the studies of cellular components (CC), molecular functions (MF), and biological processes (BP) were performed. **Supplementary Table 1** displays the biological process subcategories in which gene expression was substantially changed in response to TAZ knockdown. **Supplementary Table 1** categorizes the genes with significantly increased or decreased expression by TAZ knockdown that are involved in biological processes. Furthermore, we classified genes with significantly increased or decreased expression by TAZ knockdown into molecular functions (**Supplementary Table 2**).

### Rho GTPase protein and mRNA levels in MDA-MB-231 cells with siRNA-mediated TAZ knockdown

**Figure 4:**
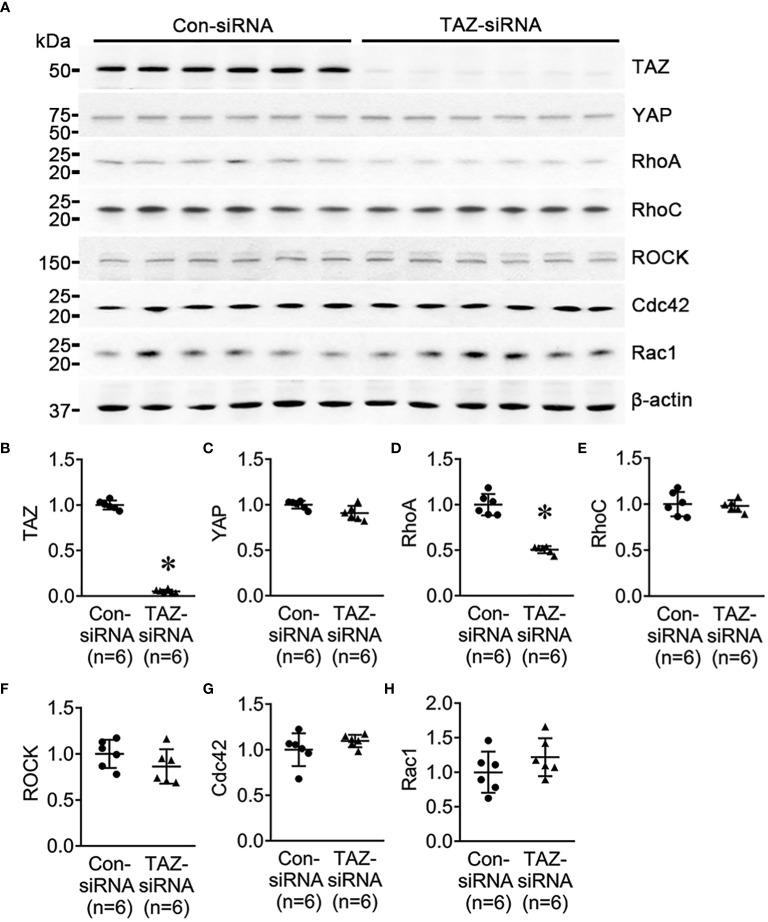
Semiquantitative immunoblotting for Rho family GTPases. Changes in Rho GTPase protein abundance in breast cancer cells treated with control-siRNA or TAZ-siRNA. **(A–H)** Semiquantitative immunoblotting of TAZ, YAP, RhoA, RhoC, ROCK, Cdc42, Rac1, and β-actin in MDA-MB-231 cells treated with control-siRNA or TAZ-siRNA. The immunoblots were reacted with antibodies against TAZ (~49 kDa), RhoA (~22 kDa), RhoC (~21 kDa), ROCK (~158 kDa), Cdc42 (~21 kDa), Rac1 (~21 kDa), and β-actin (~42 kDa).

**Figure 5:**
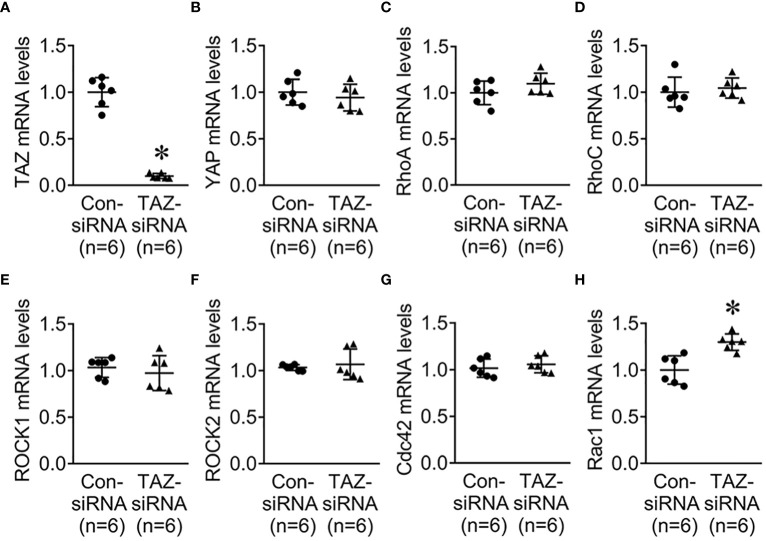
Quantitative real-time PCR for Rho family GTPases. **(A–H)** mRNA levels of TAZ, YAP, RhoA, RhoC, ROCK1, ROCK2, Cdc42, and Rac1 in MDA-MB-231 cells treated with control-siRNA or TAZ-siRNA. *P < 0.05, when compared with control-siRNA. *n* indicates the number of cell preparation in each group.

Previous studies revealed that the Rho family GTPases, RhoA, Cdc42, and Rac1, are associated with cytoskeleton rearrangements and cell motility (33, 34). Our NGS data revealed that several genes which are involved in regulation of actin dynamics and Rho GTPases were downregulated in MDA-MB-231 cells with TAZ knockdown (**Tables 3**, **4**). The alterations in the abundance of Rho GTPase proteins and mRNAs were further examined in MDA-MB-231 cells with siRNA-mediated TAZ knockdown. siRNA-mediated TAZ knockdown significantly decreased the protein abundance of TAZ (5 ± 2% of control-siRNA, **Figures 4A, B**), RhoA (50 ± 5% of control-siRNA, *P* < 0.05, **Figures 4A, D**). In contrast, YAP, ROCK, RhoC, Cdc42, and Rac1 were unchanged (**Figures 4A, C, E–H**). In addition, quantitative real-time PCR demonstrated that TAZ-siRNA mediated TAZ knockdown significantly decreased TAZ mRNA expression (10 ± 6% of control-siRNA, *P* < 0.05, **Figure 5A**). In contrast, *RAC1* mRNA levels were increased (130 ± 7% of control-siRNA, *P* < 0.05, **Figure 5H**) in TAZ-knockdown condition, whereas *YAP, RHOA, RHOC, ROCK1, ROCK2* and *CDC42* mRNA levels were unchanged (**Figures 5B–G**).

**Figure 6:**
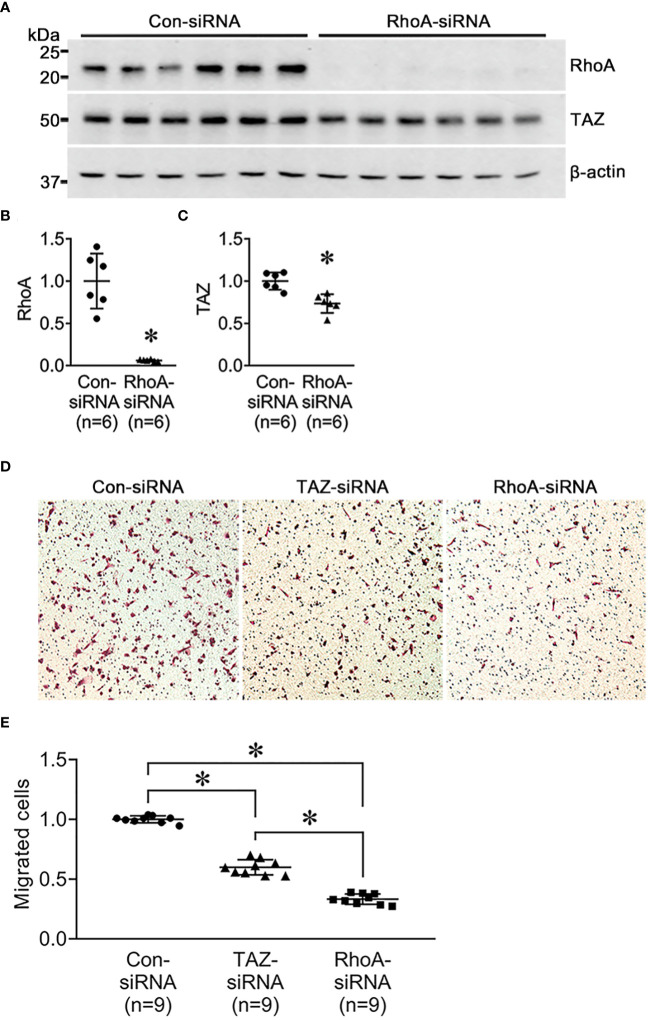
Semiquantitative immunoblotting of RhoA-siRNA-treated MDA-MB-231 cells and cell migration assay in MDA-MB-231 cells treated with control-siRNA, TAZ-siRNA, or RhoA-siRNA, respectively. **(A–D)** Changes in protein abundance of RhoA and TAZ in MDA-MB-231 cell treated with control-siRNA or RhoA-siRNA for 24 hours. *P < 0.05, when compared with control-siRNA. The immunoblots were reacted with antibodies against RhoA (~22kDa), TAZ (~49 kDa), and β-actin (~42 kDa). *n* indicates the number of cell preparation in each group. **(D, E)** Transwell cell migration assay of MDA-MB-231 cells treated with control-siRNA, TAZ-siRNA, or RhoA-siRNA treatment. The numbers of migrated cells were counted in the randomly selected fields (x100) per well. *n* indicates the number of randomly selected fields in each group. *P < 0.05.

Next, we investigated the changes in cell migration in response to siRNA-mediated RhoA knockdown. MDA-MB-231 were treated for 24 h with control-siRNA, TAZ-siRNA, or RhoA-siRNA. Immunoblot analysis demonstrated that RhoA knockdown was induced by RhoA-siRNA treatment in MDA-MB-231 cells (**Figures 6A, B**). RhoA-siRNA treatment significantly decreased the protein abundance of RhoA (6 ± 13% of control-siRNA, *P* < 0.05, **Figures 6A, B**) and TAZ (73 ± 6% of control-siRNA, *P* < 0.05, **Figures 6A, C**). The transwell migration assay demonstrated that migration of MDA-MB-231 cells was significantly decreased in response to TAZ-siRNA-mediated knockdown (59 ± 3% of control-siRNA treated cells, *P* < 0.05, **Figures 6D, E**) or RhoA-siRNA-mediated knockdown (33 ± 2% of control-siRNA treated cells, *P* < 0.05, **Figures 6D, E**).

### Rho/ROCK pathway-dependent downstream protein levels in breast cancer cells with siRNA-mediated TAZ knockdown

**Figure 7:**
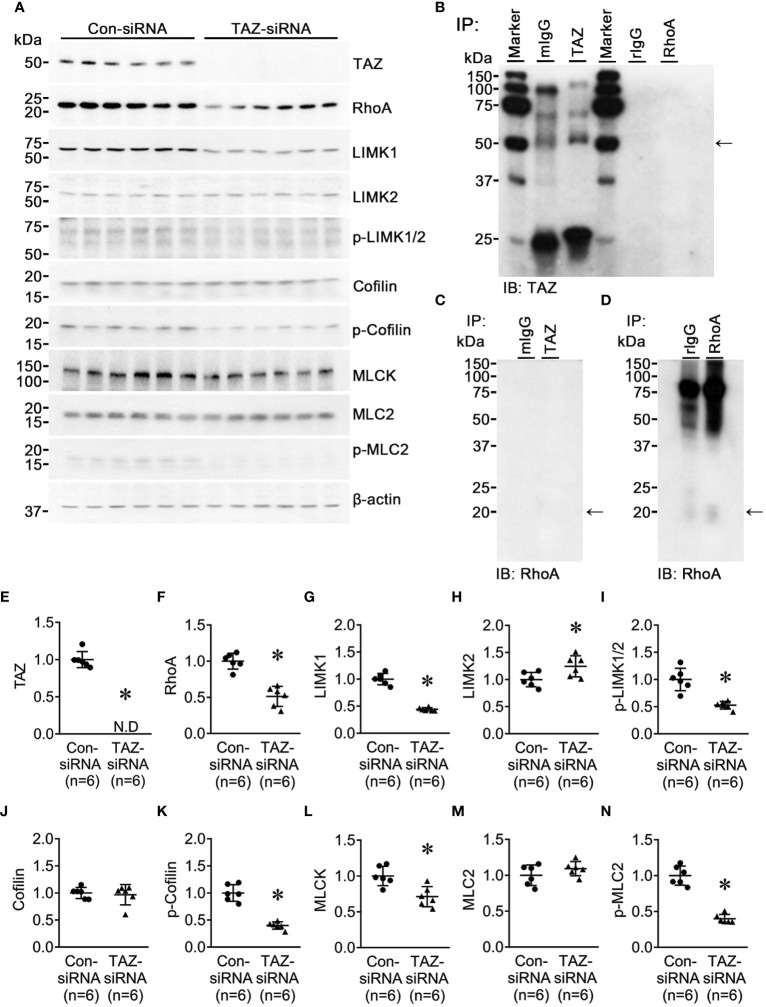
Semiquantitative immunoblotting for Rho/ROCK/LIMK and Rho/MLCK/MLC2 pathway. Changes in Rho/ROCK pathway dependent downstream protein abundance in breast cancer cells treated with control-siRNA or TAZ-siRNA. **(A, E–N)** Semiquantitative immunoblotting of TAZ, RhoA, LIMK1, LIMK2, p-LIMK, cofilin, p-cofilin, MLCK, MLC2, p-MLC2 and β-actin in MDA-MB-231 cells treated with control-siRNA or TAZ-siRNA. The immunoblots were reacted with antibodies against TAZ (~49 kDa), RhoA (~22 kDa), LIMK1 (~70 kDa), LIMK2 (~70 kDa), p-LIMK (~72 kDa), cofilin (~19 kDa), p-cofilin (~19 kDa), MLCK (~140 kDa), MLC2 (~18 kDa), p-MLC2 (~18 kDa), and β-actin (~42 kDa). **(B–D)** Co-immunoprecipitation of TAZ and RhoA. **(B)** Immunoblotting of TAZ in pull-down samples with anti-TAZ antibody in MDA-MB-231 cells. **(C, D)** Immunoblotting of RhoA in pull-down samples with anti-RhoA antibody in MDA-MB-231 cells. mIgG, pre-immune immunoglobulin G (IgG) of mouse; rIgG, pre-immune IgG of rabbit. *P < 0.05, when compared with control-siRNA. *n* indicates the number of cell preparation in each group.

LIM kinase (LIMK), activated by the small GTPase Rho and its downstream protein kinase ROCK, phosphorylates cofilin, an actin-depolymerizing factor, to regulate actin cytoskeletal reorganization. (35). An immunoprecipitation assay yielded no evidence of direct binding of TAZ and RhoA (**Figures 7B–D**). TAZ-specific siRNA treatment significantly decreased the protein abundance of TAZ (not detectable, **Figures 7A, E**), RhoA (51 ± 7% of control-siRNA, *P* < 0.05, **Figurse 7A, F**), LIMK1 (44 ± 4% of control-siRNA, *P* < 0.05, **Figures 7A, G**), phosphorylated LIMK1 at threonine 508/LIMK2 at threonine 505 (p-LIMK1/2, 52 ± 9% of control-siRNA, *P* < 0.05, **Figures 7A, I**) and phosphorylated cofilin at serine 3 (40 ± 7% of control-siRNA, *P* < 0.05, **Figures 7A, K**). In contrast, LIMK2 protein abundance was increased (124 ± 10% of control-siRNA, *P* < 0.05, **Figures 7A, H**), and cofilin and MLC2 were unchanged (**Figures 7A, J, M**). Next, we studied whether TAZ knockdown affects the expression and phosphorylation of myosin light chain kinase (MLCK), which phosphorylates myosin light chain (MLC) and plays a role in stress fiber assembly and focal adhesion formation. siRNA-mediated TAZ knockdown was associated with significantly decreased protein abundance of MLCK (71 ± 8% of control-siRNA, *P* < 0.05, **Figures 7A, L**) and phosphorylated MLC2 at serine 19 (39 ± 6% of control-siRNA, *P* < 0.05, **Figures 7A, N**).

### Altered stress fiber formation and expression of myosin light chain kinase in breast cancer cells with siRNA-mediated TAZ knockdown

**Figure 8:**
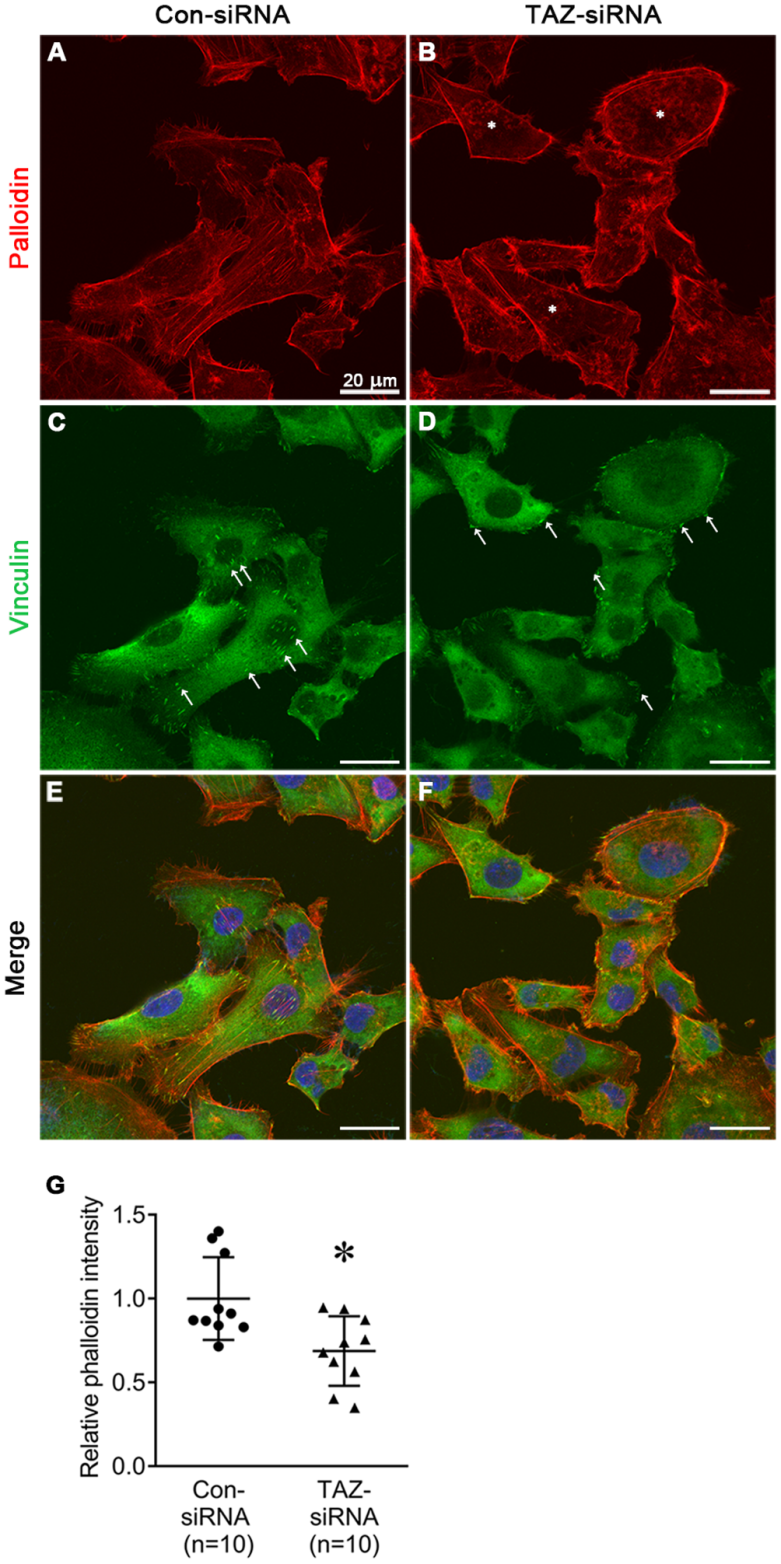
Fluorescence microscopic examination of stress fibers and immunolabeling of vinculin in MDA-MB-231 cells treated with control-siRNA **(A, C, E)** or TAZ-siRNA **(B, D, F)**. Nuclei were labeled with 4’,6-Diamidino-2-Phenylindole (DAPI). Scale bars, 20 μm. **(G)** The quantification of phalloidin intensity. Relative phalloidin intensity was measured by averaging the fluorescence intensity of 5 randomly selected regions of interest in MDA-MB-231 cells. *n* indicates the number of cells in each group. *P < 0.05.

**Figure 9:**
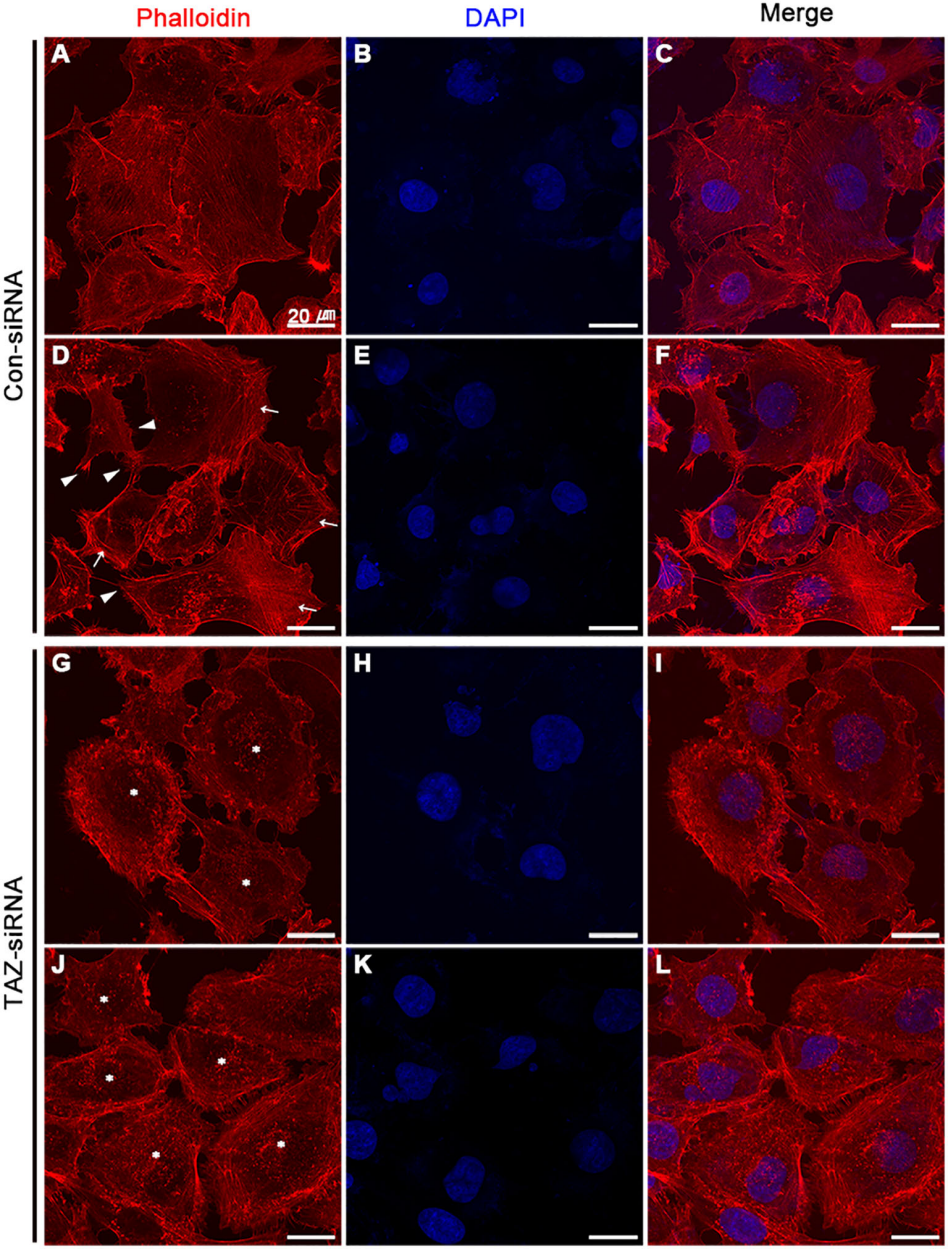
Fluorescence microscopic examination of stress fibers and immunolabeling of vinculin in MDA-MB-231 cells treated with control-siRNA **(A–F)** or TAZ-siRNA **(G–L)**. Nuclei were labeled with 4’,6-Diamidino-2-Phenylindole (DAPI). Scale bars, 20 μm.

We examined the changes in the stress fiber formation in MDA-MB-231 cells in response to TAZ knockdown. Stress fibers were stained using phalloidin, demonstrated in red (**Figures 8**, **9**), and vinculin was immunolabeled in green as a marker of focal adhesion (arrows in **Figures 8C, D**). In control-siRNA-treated MDA-MB-231 cells, stress fibers were observed to be widely distributed intracellularly in both the central and peripheral regions of the cells (**Figures 8A, E**, **9A, C, D, F**), and immunolabeling of vinculin was observed at the ends of the stress fibers (arrows in **Figure 8C**). Moreover, stress fibers were more abundantly expressed on the rear side of the cells (arrows in **Figure 9D**), and lamellipodia were predominantly detected on the front side (arrowheads in **Figure 9D**). On the contrary, TAZ-siRNA-treated MDA-MB-231 cells exhibited a decrease in the labeling of stress fibers, observed mainly in the central regions of the cells (asterisks in **Figures 8B**, **9H, K**) and the immunolabeling of vinculin (focal adhesion) was mainly observed at the periphery of the cells (arrows in **Figure 8D**). Furthermore, the disassembly of stress fibers was observed in MDA-MB-231 cells treated with TAZ-siRNA (**Figures 8B, F**, **9G, I, J, L**). We quantified the intensity of phalloidin (stress fiber) staining using ImageJ. The five regions of interest per cell were randomly selected and measured. Fluorescence intensity was measured in 10 cells per group, and a significant decrease in the phalloidin intensity was demonstrated in TAZ-siRNA treated MDA-MB-231 cells (68 ± 10% of control-siRNA, *P* < 0.05, **Figure 8G**).

## Discussion

The characteristics of metastatic breast cancer cells are diverse, including EMT and increased AQP5 expression (14, 36–38). In particular, the metastatic breast cancer cells in human patients display an increased level of TAZ expression (39, 40). In the present study, we demonstrated that TAZ knockdown inhibited the migration of highly invasive triple-negative breast cancer cells (MDA-MB-231) by reducing the protein abundance of RhoA and Rho-dependent kinases, particularly LIMK1 and MLCK. The downregulation of RhoA and Rho-dependent kinases in response to TAZ knockdown is likely to play a role in the reduction of stress fibers in the central region of the cells, disassembly of stress fibers, and diffuse focal adhesions throughout the cell periphery, resulting in a decrease in cell migration. Although the findings were obtained purely from invasive triple-negative breast cancer cells, the results may suggest that TAZ could be investigated as a therapeutic target for the inhibition of the migration of breast cancer cells and metastasis.

### Decreased cell migration in the invasive breast cancer cells (MCF-7 and MDA-MB-231 cells) in response to TAZ knockdown

We analyzed two different invasive breast cancer cell lines (MCF-7 and MDA-MB-231 cells) with distinct morphological characteristics. Even though there was less TAZ in MCD-7 cells than in MDA-MB-231 cells, siRNA effectively reduced TAZ levels in both cell lines. Moreover, TAZ knockdown decreased cell migration in both MCF-7 and MDA-MB-231 cells. However, we conducted subsequent experiments exclusively with MDA-MB-231 cells due to the limited cell proliferation and migration observed in MCF-7 cells. Furthermore, previous studies demonstrated an association between higher levels of the TAZ protein and aggressive and invasive metastatic breast cancer cells (39, 41).

### TAZ knockdown did not induce EMT in MDA-MB-231 cells

Several previous studies have demonstrated that TAZ induces EMT in various types of cancer (42–44). We examined whether TAZ knockdown affects the changes in EMT makers in MDA-MB-231 cells. As shown in **Figure 2**, an increase in fibronectin abundance may indicate an increase in the mesenchymal characteristics of cancer cells. On the other hand, the changes in E-cadherin, occludin, N-cadherin, and vimentin did not exhibit the typical pattern of EMT progression.

Then, we examined the effects of TGF-β treatment on cell migration under TAZ knockdown. An experiment was conducted to determine whether inducing TAZ knockdown in MDA-MB-231 cells, which already exhibit mesenchymal characteristics, could inhibit the morphological changes and cell migration induced by TGF-β treatment. Interestingly, TGF-β treatment resulted in a more pronounced reduction in the protein abundance of TAZ in the MDA-MB-231 cells with siRNA-mediated TAZ knockdown. Moreover, TGF-β treatment increased fibronectin abundance and enhanced cell migration. Furthermore, the increased fibronectin abundance induced by either TAZ knockdown or TGF-β treatment (possibly the mesenchymal characteristics of cancer cells) did not contribute to breast cancer cell migration, as TAZ knockdown decreased cell migration, while TGF-β treatment had the opposite effect despite the presence of TAZ knockdown (**Figure 3**). Therefore, TAZ knockdown in MDA-MB-231 cells was incapable of acquiring epithelial cell characteristics. Moreover, it did not impede TGF-β-induced cell migration, suggesting that TAZ knockdown reduces breast cancer cell migration independently of EMT.

### Decrease in RhoA, phosphorylated LIMK1, and phosphorylated cofilin in response to TAZ knockdown in MDA-MB-231 cells

The NGS analysis of control-siRNA-treated and TAZ-siRNA-treated MDA-MB-231 cells showed substantial changes in the expression levels of genes encoding small GTPases, Rho-GAPs, and Rho-GEFs, all of which are involved in the organization of the actin cytoskeleton. The small GTPases RhoA and RhoC exhibited the highest levels of expression within the Rho family, as demonstrated by NGS analysis. RhoA tended to decrease with TAZ knockdown, while RhoC showed no difference. Semiquantitative immunoblotting in **Figure 4** revealed a significant decrease in RhoA protein levels in TAZ-siRNA-treated MDA-MB-231 cells. We tested other small GTPases (Cdc42 and Rac1), but there were no differences in mRNA or protein levels. Rho-dependent kinase (ROCK) is a downstream effector of RhoA and RhoC, which regulates actomyosin assembly, actomyosin contractility, and reorganization of the actin cytoskeleton by regulating the phosphorylation of downstream kinases or phosphatases (45). The binding of active RhoA to ROCK initiates a phosphorylation cascade that controls the dynamics of cytoskeletal actin (46, 47). ROCK mRNA and protein levels stayed the same in MDA-MB-231 cells treated with TAZ-siRNA. However, the lower expression of RhoA may have an effect on the kinases that follow in the RhoA-dependent pathway. In particular, LIM kinase (LIMK), activated by the small GTPase Rho and its downstream protein kinase ROCK, phosphorylates cofilin, an actin-depolymerizing factor, to regulate actin cytoskeletal reorganization. (35). Notably, we demonstrated that RhoA and LIMK1 were significantly decreased by TAZ knockdown in MDA-MB-231 cells. As shown in **Figure 7**, the TAZ knockdown resulted in a significant decrease in the abundance of phosphorylated LIMK (LIMK1 at threonine 508 and LIMK-2 at threonine 505) at ~72 kDa, which represents an activated form of LIMK. Furthermore, in MDA-MB-231 cells treated with TAZ-siRNA, phosphorylated cofilin at serine 3 was significantly reduced, thereby enhancing cofilin-induced actin depolymerization (48). The findings indicate that TAZ knockdown reduces RhoA protein expression, which subsequently causes the downregulation of the RhoA/ROCK/LIMK1/cofilin pathway, resulting in a change in actin dynamics. In accordance with this observation, the migration of MDA-MB-231 cells was found to be diminished upon RhoA knockdown. On the other hand, the results of the immunoprecipitation assay did not provide any evidence of direct binding between TAZ and RhoA. Therefore, it is necessary for future research to investigate the underlying mechanisms by which TAZ controls the expression of RhoA and downstream kinases.

We also examined the changes in actin dynamics by phalloidin staining and immunofluorescence labeling. We observed the changes in the expression of vinculin, a highly expressed anchor protein involved in focal adhesion that interacts with F-actin and the membrane. Vinculin (**Figure 8**) was mainly observed at the end of stress fibers distributed in both the periphery and central region of the control-siRNA-treated MDA-MB-231 cells. On the other hand, in response to TAZ knockdown, the cells showed exclusive vinculin localization at the periphery, with no apparent stress fiber staining in the central region. These findings are consistent with actin depolymerization that occurred due to decreased phosphorylation of cofilin.

### Decreased MLCK and phosphorylated MLC in response to TAZ knockdown in MDA-MB-231 cells

Rho-kinase and MLCK are known to regulate the organization and contraction of the stress fibers in the central region of the cells and the organization of focal adhesion (49–51). siRNA-mediated TAZ knockdown in MDA-MB-231 cells banished the stress fibers and focal adhesion in the central region of the cells. We looked at the Rho/ROCK/MLCK pathway, which controls the formation and contractility of central stress fibers, to see if TAZ knockdown changed the phosphorylation of MLCK and MLC. Consistently, in TAZ-siRNA-treated MDA-MB-231 cells, the protein abundance of MLCK and phosphorylated MLC2 at serine 19 was significantly decreased, but the total abundance of MLC2 was unchanged.

In summary, the findings observed in response to TAZ knockdown demonstrated that a decrease in LIMK1 and phosphorylated cofilin caused actin depolymerization, while a reduction in MLCK and phosphorylated MLC2 led to the disassembly of stress fibers and focal adhesions, ultimately resulting in a decrease in breast cancer cell migration.

## Data availability statement

The dataset presented in the study can be found in online repositories (NCBI's Gene Expression Omnibus) and are accessible through GEO Series accession number GSE264547 (https://www.ncbi.nlm.nih.gov/geo/query/acc.cgi?acc=GSE264547).

## Ethics statement

Ethical approval was not required for the studies on humans in accordance with the local legislation and institutional requirements because only commercially available established cell lines were used.

## Author contributions

HC: Writing – review & editing, Writing – original draft, Visualization, Validation, Methodology, Investigation, Formal analysis, Data curation, Conceptualization. HJ: Writing – review & editing, Writing – original draft, Visualization, Validation, Methodology, Investigation, Formal analysis, Data curation, Conceptualization. MK: Writing – review & editing, Writing – original draft, Visualization, Validation, Methodology, Investigation, Formal analysis, Conceptualization. TK: Writing – review & editing, Writing – original draft, Visualization, Validation, Supervision, Software, Resources, Project administration, Methodology, Investigation, Funding acquisition, Formal analysis, Data curation, Conceptualization.
