# Supplementary material for: TAZ is involved in breast cancer cell migration via regulating actin dynamics
Source: Front Oncol. 2024 May 7;14:1376831. doi: 10.3389/fonc.2024.1376831 (PMC11106448; doi:10.3389/fonc.2024.1376831)
Supplement: Supplementary file 1 [file Table_1.docx]

| **Supplementary Table 1. GO biological process related with TAZ knockdown** | | | |  |  |  |
| --- | --- | --- | --- | --- | --- | --- |
| Term id | Term name of biological process | p-value | The number of genes | | The number of upregulated genes | The number of downregulated genes |
| GO:0065007 | biological regulation | 0.03497273 | 106 | | 56 | 50 |
| GO:0050789 | regulation of biological process | 0.018864423 | 103 | | 55 | 48 |
| GO:0050794 | regulation of cellular process | 0.004364412 | 99 | | 51 | 48 |
| GO:0050896 | response to stimulus | 1.01973E-05 | 94 | | 44 | 50 |
| GO:0032501 | multicellular organismal process | 5.17116E-06 | 84 | | 42 | 42 |
| GO:0051716 | cellular response to stimulus | 0.00011876 | 79 | | 36 | 43 |
| GO:0051179 | localization | 5.17116E-06 | 77 | | 43 | 34 |
| GO:0023052 | signaling | 1.01973E-05 | 76 | | 36 | 40 |
| GO:0007154 | cell communication | 2.29746E-05 | 75 | | 35 | 40 |
| GO:0007165 | signal transduction | 1.77976E-05 | 71 | | 31 | 40 |
| GO:0032502 | developmental process | 0.000180414 | 70 | | 32 | 38 |
| GO:0048518 | positive regulation of biological process | 0.000216833 | 69 | | 28 | 41 |
| GO:0048856 | anatomical structure development | 4.22106E-05 | 68 | | 32 | 36 |
| GO:1901564 | organonitrogen compound metabolic process | 0.004632243 | 65 | | 28 | 37 |
| GO:0048522 | positive regulation of cellular process | 0.000347562 | 64 | | 26 | 38 |
| GO:0007275 | multicellular organism development | 1.09993E-05 | 62 | | 29 | 33 |
| GO:0019538 | protein metabolic process | 0.000442496 | 62 | | 26 | 36 |
| GO:0044260 | cellular macromolecule metabolic process | 0.001476172 | 62 | | 26 | 36 |
| GO:0042221 | response to chemical | 3.19914E-06 | 61 | | 24 | 37 |
| GO:0048731 | system development | 3.8698E-06 | 60 | | 29 | 31 |
| GO:0030154 | cell differentiation | 1.01973E-05 | 57 | | 24 | 33 |
| GO:0048869 | cellular developmental process | 1.06578E-05 | 57 | | 24 | 33 |
| GO:0044267 | cellular protein metabolic process | 0.00087825 | 56 | | 23 | 33 |
| GO:0048519 | negative regulation of biological process | 0.033587359 | 56 | | 25 | 31 |
| GO:0006810 | transport | 0.000812641 | 55 | | 30 | 25 |
| GO:0051234 | establishment of localization | 0.001547068 | 55 | | 30 | 25 |
| GO:0048513 | animal organ development | 2.55086E-06 | 54 | | 26 | 28 |
| GO:0051239 | regulation of multicellular organismal process | 4.30906E-09 | 52 | | 26 | 26 |
| GO:0023051 | regulation of signaling | 3.19914E-06 | 52 | | 24 | 28 |
| GO:0010646 | regulation of cell communication | 3.8698E-06 | 51 | | 23 | 28 |
| GO:0048583 | regulation of response to stimulus | 9.92139E-05 | 51 | | 21 | 30 |
| GO:0048523 | negative regulation of cellular process | 0.010063645 | 51 | | 22 | 29 |
| GO:0065008 | regulation of biological quality | 9.09421E-05 | 50 | | 25 | 25 |
| GO:0043412 | macromolecule modification | 0.000907296 | 48 | | 19 | 29 |
| GO:0006464 | cellular protein modification process | 0.000504446 | 47 | | 19 | 28 |
| GO:0036211 | protein modification process | 0.000504446 | 47 | | 19 | 28 |
| GO:0009966 | regulation of signal transduction | 1.52907E-05 | 45 | | 17 | 28 |
| GO:0009893 | positive regulation of metabolic process | 0.002162629 | 45 | | 16 | 29 |
| GO:0007166 | cell surface receptor signaling pathway | 1.18584E-05 | 44 | | 16 | 28 |
| GO:0010033 | response to organic substance | 4.92242E-05 | 44 | | 17 | 27 |
| GO:0009653 | anatomical structure morphogenesis | 1.12849E-05 | 43 | | 21 | 22 |
| GO:0010604 | positive regulation of macromolecule metabolic process | 0.001462957 | 43 | | 15 | 28 |
| GO:0009605 | response to external stimulus | 4.45104E-05 | 42 | | 19 | 23 |
| GO:0070887 | cellular response to chemical stimulus | 0.000207444 | 42 | | 16 | 26 |
| GO:0050793 | regulation of developmental process | 7.64104E-06 | 41 | | 18 | 23 |
| GO:0006950 | response to stress | 0.023408939 | 41 | | 16 | 25 |
| GO:0035556 | intracellular signal transduction | 6.32233E-05 | 40 | | 15 | 25 |
| GO:0032879 | regulation of localization | 0.000119033 | 40 | | 17 | 23 |
| GO:0065009 | regulation of molecular function | 0.001026001 | 40 | | 14 | 26 |
| GO:0031325 | positive regulation of cellular metabolic process | 0.003189912 | 40 | | 13 | 27 |
| GO:0051173 | positive regulation of nitrogen compound metabolic process | 0.002118278 | 39 | | 12 | 27 |
| GO:0009888 | tissue development | 2.55086E-06 | 38 | | 16 | 22 |
| GO:0007399 | nervous system development | 6.34441E-05 | 38 | | 20 | 18 |
| GO:0006928 | movement of cell or subcellular component | 1.01973E-05 | 37 | | 18 | 19 |
| GO:0051246 | regulation of protein metabolic process | 0.000287208 | 37 | | 8 | 29 |
| GO:0006796 | phosphate-containing compound metabolic process | 0.001367993 | 37 | | 9 | 28 |
| GO:0006793 | phosphorus metabolic process | 0.00153871 | 37 | | 9 | 28 |
| GO:0040011 | locomotion | 3.8698E-06 | 36 | | 17 | 19 |
| GO:0003008 | system process | 0.000136471 | 35 | | 18 | 17 |
| GO:0032268 | regulation of cellular protein metabolic process | 0.000392566 | 35 | | 7 | 28 |
| GO:0071310 | cellular response to organic substance | 0.000451839 | 35 | | 13 | 22 |
| GO:0008283 | cell population proliferation | 3.40163E-05 | 34 | | 12 | 22 |
| GO:0048584 | positive regulation of response to stimulus | 0.000180078 | 34 | | 8 | 26 |
| GO:0051240 | positive regulation of multicellular organismal process | 1.55364E-06 | 33 | | 13 | 20 |
| GO:0048468 | cell development | 0.000219251 | 33 | | 14 | 19 |
| GO:0050790 | regulation of catalytic activity | 0.001337013 | 33 | | 9 | 24 |
| GO:0002376 | immune system process | 0.01450789 | 33 | | 14 | 19 |
| GO:0048870 | cell motility | 1.1278E-05 | 32 | | 15 | 17 |
| GO:0051674 | localization of cell | 1.1278E-05 | 32 | | 15 | 17 |
| GO:2000026 | regulation of multicellular organismal development | 2.55086E-06 | 31 | | 14 | 17 |
| GO:0022008 | neurogenesis | 2.43055E-05 | 31 | | 15 | 16 |
| GO:0042127 | regulation of cell population proliferation | 2.74918E-05 | 31 | | 9 | 22 |
| GO:0007267 | cell-cell signaling | 2.80019E-05 | 31 | | 13 | 18 |
| GO:0016310 | phosphorylation | 0.000190189 | 31 | | 6 | 25 |
| GO:0048699 | generation of neurons | 8.22882E-06 | 30 | | 15 | 15 |
| GO:0051128 | regulation of cellular component organization | 0.005967371 | 30 | | 12 | 18 |
| GO:0051094 | positive regulation of developmental process | 3.8698E-06 | 29 | | 11 | 18 |
| GO:0009967 | positive regulation of signal transduction | 3.80011E-05 | 29 | | 6 | 23 |
| GO:0010647 | positive regulation of cell communication | 0.000225454 | 29 | | 6 | 23 |
| GO:0023056 | positive regulation of signaling | 0.000231812 | 29 | | 6 | 23 |
| GO:0030182 | neuron differentiation | 2.07454E-05 | 28 | | 13 | 15 |
| GO:0019220 | regulation of phosphate metabolic process | 2.54118E-05 | 28 | | 6 | 22 |
| GO:0051174 | regulation of phosphorus metabolic process | 2.55976E-05 | 28 | | 6 | 22 |
| GO:0016477 | cell migration | 5.48791E-05 | 28 | | 13 | 15 |
| GO:0031399 | regulation of protein modification process | 9.92139E-05 | 28 | | 4 | 24 |
| GO:0045595 | regulation of cell differentiation | 0.00012171 | 28 | | 14 | 14 |
| GO:0006811 | ion transport | 0.000121816 | 28 | | 17 | 11 |
| GO:0006468 | protein phosphorylation | 0.000171344 | 28 | | 6 | 22 |
| GO:0007155 | cell adhesion | 0.00016158 | 27 | | 12 | 15 |
| GO:0008219 | cell death | 0.012088677 | 27 | | 9 | 18 |
| GO:0009887 | animal organ morphogenesis | 5.17116E-06 | 26 | | 14 | 12 |
| GO:0042325 | regulation of phosphorylation | 3.35608E-05 | 26 | | 5 | 21 |
| GO:0051247 | positive regulation of protein metabolic process | 0.000409482 | 26 | | 3 | 23 |
| GO:0044093 | positive regulation of molecular function | 0.000748518 | 26 | | 6 | 20 |
| GO:0009719 | response to endogenous stimulus | 0.0007511 | 26 | | 11 | 15 |
| GO:0010941 | regulation of cell death | 0.000998293 | 26 | | 9 | 17 |
| GO:1902531 | regulation of intracellular signal transduction | 0.001981048 | 26 | | 8 | 18 |
| GO:0051049 | regulation of transport | 0.002506769 | 26 | | 11 | 15 |
| GO:0048666 | neuron development | 1.77976E-05 | 25 | | 11 | 14 |
