# Supplementary material for: TAZ is involved in breast cancer cell migration via regulating actin dynamics
Source: Front Oncol. 2024 May 7;14:1376831. doi: 10.3389/fonc.2024.1376831 (PMC11106448; doi:10.3389/fonc.2024.1376831)
Supplement: Supplementary file 2 [file Table_2.docx]

| **Supplementary Table 2. Gene expressions in GO: biological process and molecular function** | | | | | | |  |
| --- | --- | --- | --- | --- | --- | --- | --- |
| Term_id | GO | Term_name | The number of related genes | P-value | Upregulated genes | Downregulated genes | |
| GO:0032879 | Biological process | Regulation of localization | 40 | 0.000119033 | ADORA1, CACNA1H, CAV3, CNIH3, CYGB, GLI1, GRIN2D, IGFBP5, NKAIN2, NOS2, RAPGEF4, RSAD2, SCN2A, SEMA6B, SPOCK3, TRPC3 | ADORA2A, ADRB2, ANKRD1, CCN1, CD33, CLDN1, DKK1, EDN1, FGF1, KISS1, KLF15, LAMA1, LPAR1, NCKAP1L, RELN, SMPD3, TGFB2, TNFSF18 | |
| GO:0048870 | Biological process | Cell motility | 32 | 1.1278E-05 | ASB2, BCL11B, CYGB, EPHB1, GLI1, IGFBP5, ITGB8, KIAA0319, SEMA6B, SLIT1, TPTE2 | CCBE1, CCN2, CLDN1, FAM71D, FGF1, LAMA1, LPAR1, NCKAP1L, PDGFB, SMPD3, TNFSF18, VAX1 | |
| GO:0016477 | Biological process | Cell migration | 28 | 5.48791E-05 | ADORA1, ASB2, BCL11B, CYGB, EPHB1, ITGB8, KIAA0319, NTNG2, SDC3, SEMA6B | CCBE1, CCN1, CCN2, CLDN1, FGF1, LAMA1, LPAR1, NCKAP1L, PDGFB, RELN, SMPD3, TGFB2, TNFSF18, VAX1 | |
| GO:0040012 | Biological process | Regulation of locomotion | 22 | 8.49193E-05 | ADORA1, CYGB, GLI1, IGFBP5, NTNG2, SEMA6B, SLIT1 | ADORA2A, CCBE1, CCN1, CLDN1, FGF1, LAMA1, LPAR1, PDGFB, RELN, SMPD3, TNFSF18 | |
| GO:2000145 | Biological process | Regulation of cell motility | 20 | 0.000347562 | GLI1, IGFBP5, NTNG2, SEMA6B, SPOCK3 | CCBE1, CCN1, CLDN1, EDN1, FGF1, LAMA1, LPAR1, NCKAP1L, RELN, TGFB2, TNFSF18 | |
| GO:0030334 | Biological process | Regulation of cell migration | 19 | 0.00045874 | ADORA1, CYGB, GLI1, IGFBP5, NTNG2, SEMA6B | CCBE1, CCN1, CLDN1, EDN1, FGF1, LAMA1, NCKAP1L, PDFGB, SMPD3, TGFB2, TNFSF18 | |
| GO:0030335 | Biological process | Positive regulation of cell migration | 14 | 0.000548286 | GL1, IGFBP5, SEMA6B | CCBE1, CCN1, CLDN1, EDN1, FGF1, NCKAP1L, RELN, TGFB2, TNFSF18 | |
| GO:2000147 | Biological process | Positive regulation of cell motility | 14 | 0.000826395 | GLI1, IGFBP5, SEMA6B | CCBE1, CCN1, CLDN1, EDN1, FGF1, LPAR1, NCKAP1L, RELN | |
| GO:0040017 | Biological process | Positive regulation of locomotion | 14 | 0.001033218 | GLI1, SEMA6B | CCBE1, CCN1, CLDN1, EDN1, FGF1, LPAR1, NCKAP1L, PDGFB, RELN, TGFB2, TNFSF18 | |
| GO:0040013 | Biological process | Negative regulation of locomotion | 7 | 0.041405263 | CYGB, IGFBPR5, SEMA6B, SLIT1, SPOCK3 | ADORA2A | |
| GO:0031032 | Biological process | Actomyosin structure organization | 6 | 0.023025154 | - | ANKRD1, CCN2, EDN1, LPAR1, MYPN | |
| GO:0110020 | Biological process | Regulation of actomyosin structure organization | 4 | 0.035130758 | - | CCN2, EDN1, LPAR1 | |
| GO:0098772 | Molecular function | Molecular function regulator activity | 28 | 0.044505385 | ACAP1, CST2, LYNX1, RAPGEF4, SEMA6B, SPOCK3, WNT2B | ADRB2, BIRC3, CALCB, CCN2, DKK1, EDN1, FGF1, IGF2, IL32, INSL4, NCKAP1L, NGF, NUPR1, PDFGB, PIK3R5, RGS7, TGFB2, TNFSF15, TNFSF18 | |
| GO:0005102 | Molecular function | Signaling receptor binding | 27 | 0.004352481 | ADORA1, ATP2B2, BDKRB2, ITGB8, LYNX1, SEMA6B, SLIT1, SNED1, WNT2B | ADORA2A, CALCB, CCN1, CCN2, DKK1, EDN1, FGF1, IGF2, IL32, INSL4, ITGB8, KISS1, LAMA1, NGF, PDGFB, RELN, TGFB2, TNFSF15, TNFSF18 | |
| GO:0005509 | Molecular function | Calcium ion binding | 16 | 0.011829243 | ATP2B2, LRP1B, MMP13, NCALD, PCDHGA4, PCDHGA10, PCDHB2, PRSS2, SLIT1, SNED1, SPOCK3 | ANXA8, CCBE1 | |
| GO:0030545 | Molecular function | Signaling receptor regulator activity | 16 | 0.001044889 | LYNX1, SEMA6B, WNT2B | CALCB, CCN2, DKK1, EDN1, FGF1, IGF2, IL32, INSL4, NGF, PDGF, TGFB2, TNFSF15, TNFSF18 | |
| GO:0048018 | Molecular function | Receptor ligand activity | 15 | 0.001044889 | SEMA6B, WNT2B | CALCB, CCN2, DKK1, EDN1, FGF1, IGF2, IL32, INSL4, NGF, PDGFB, TGFB2, TNFSF15, TNFSF18 | |
| GO:0030546 | Molecular function | Signaling receptor activator activity | 15 | 0.001044889 | SEMA6B, WNT2B | CALCB, CCN2, DKK1, EDN1, FGF1, IGF2, IL32, INSL4, NGF, PDGFB, TGFB2, TNFSF15, TNFSF18 | |
| GO:0008083 | Molecular function | Growth factor activity | 7 | 0.021369721 | - | CCN2, DKK1, FGF1, IGF2, NFG, PDGFB, TGFB2 | |
| GO:0001609 | Molecular function | G protein-coupled adenosine receptor activity | 2 | 0.042109248 | ADORA1 | ADORA2A | |
